# Valuing productivity loss due to absenteeism: firm-level evidence from a Canadian linked employer-employee survey

**DOI:** 10.1186/s13561-016-0138-y

**Published:** 2017-01-19

**Authors:** Wei Zhang, Huiying Sun, Simon Woodcock, Aslam H. Anis

**Affiliations:** 1 Centre for Health Evaluation and Outcome Sciences, St. Paul’s Hospital, 588-1081 Burrard Street, Vancouver, BC V6Z1Y6 Canada; 2 School of Population and Public Health, University of British Columbia, 2206 East Mall, Vancouver, BC V6T1Z3 Canada; 3 Department of Economics, Simon Fraser University, 8888 University Drive, Burnaby, BC V5A 1S6 Canada

**Keywords:** Productivity loss, Absenteeism, Marginal productivity, Wage, Teamwork, Valuation, J31, D24, I12, I15

## Abstract

In health economic evaluation studies, to value productivity loss due to absenteeism, existing methods use wages as a proxy value for marginal productivity. This study is the first to test the equality between wage and marginal productivity losses due to absenteeism separately for team workers and non-team workers. Our estimates are based on linked employer-employee data from Canada. Results indicate that team workers are more productive and earn higher wages than non-team workers. However, the productivity gap between these two groups is considerably larger than the wage gap. In small firms, employee absenteeism results in lower productivity and wages, and the marginal productivity loss due to team worker absenteeism is significantly higher than the wage loss. No similar wage-productivity gap exists for large firms. Our findings suggest that productivity loss or gain is most likely to be underestimated when valued according to wages for team workers. The findings help to value the burden of illness-related absenteeism. This is important for economic evaluations that seek to measure the productivity gain or loss of a health care technology or intervention, which in turn can impact policy makers’ funding decisions.

**Electronic supplementary material:**

The online version of this article (doi:10.1186/s13561-016-0138-y) contains supplementary material, which is available to authorized users.

## Introduction

It is still under debate whether we should take account of productivity gains or losses from a health care intervention in economic evaluation studies [1, 2]. Cost-effectiveness studies, for example, are routinely used to determine the eligibility of health technologies such as pharmaceuticals for coverage under national or provincial health plans. The inclusion of productivity losses in such analyses would have a significant influence on determinations of cost-effectiveness, leading to different resource allocation decisions. Krol et al. find that accounting for productivity costs can either increase or decrease the incremental cost-effectiveness ratio (ICER) between treatment arms [3, 4]. Thus, cost-effectiveness studies that account for productivity losses are useful in identifying interventions with a potentially broad impact, and do not necessarily lower the ICERs of an intervention.

Despite robust arguments in favour of including productivity loss in evaluation studies [3–6], current methods to value productivity loss are limited. Existing methods usually quantify productivity loss using wages as a proxy for marginal productivity [1, 7, 8]. However, wages may not equal marginal productivity for many reasons, making it a poor proxy and reducing the accuracy of estimated productivity loss. In imperfect labour markets, wages may not equal marginal productivity due to inequities, such as race or gender discrimination, whereby an identifiable group routinely receives lower wages. More commonly, risk-averse workers might willingly accept a wage below their marginal productivity in exchange for job security, e.g. allowances for sick days [9, 10].

A wedge between a worker’s wage and marginal productivity may also arise if a job involves team production or if the firm output is time-sensitive [9, 11]. Pauly et al. presented a general model demonstrating that when there is a team production and substantial team-specific human capital, the value of lost output to the firm from an absence will exceed the daily wage of the absent worker and could be as large as the total output of the team [9]. Similarly, the cost of an absence will exceed the wage when a firm incurs a penalty if it misses an output target due to the absence. In both situations, the productivity loss could be reduced if replacements are found who are either inexpensive or are close substitutes for the absent worker.

Although there are many reasons that wage may not equal marginal productivity, there is still lack of empirical evidence on their equality with regard to absenteeism and team participation. This is the first study to empirically test the wage and marginal productivity losses due to absenteeism and measure the multiplicative effect of absenteeism for team workers. This study examines the theoretical implications on the relationship between wages and productivity when a job is involved in team production. Its findings will help determine whether wages can be used as a precise proxy of marginal productivity in estimating productivity loss due to illness-related absenteeism. In addition, we use a unique employer-employee data, the Workplace and Employee Survey (WES). The advantage of these data is that they contain information on a firm’s output, capital, materials, other expenditures, payroll, and industry as well as its workers’ age, sex, education, occupation, team participation status and absenteeism. The availability of such data allows us to test the equality of wage and marginal productivity for groups of workers with different characteristics. The WES is one of only a few linked employer-employee databases worldwide and the only one for Canada. Furthermore, we conduct robustness checks using alternative specifications and dropping some of the assumptions. We find that our estimates of wage and marginal productivity losses due to absenteeism appear relatively robust and reasonable. We also divide the full sample into small firm and large firms and examine whether our estimates vary by firm size.

The remainder of this paper is organized as follows. Section 2 contains the conceptual framework and a short review of related studies. In section 3, we present our empirical specification. Section 4 describes our data and defines the main variables. In section 5, we present our findings and parameter estimates. Section 6 summarizes our findings and their implications for economic evaluators.

## Background

### Conceptual framework

A large literature has documented substantial wage differentials on the basis of firm size [12, 13], industry [14–16], group or non-group work [17, 18], union and non-union contracts [19, 20], business cycle [21, 22], competitiveness of the industry [23, 24], and government regulation [25, 26]. These wage gaps are conventionally estimated from a wage regression using individual-level data. Without an independent measure of worker productivity, however, it is difficult to determine whether these estimated wage differentials reflect productivity differentials or other factors such as wage discrimination [27, 28]. Hellerstein et al. have developed a framework to simultaneously estimate firm-level wage equations and production functions on population-based datasets that link employees’ input to their employers’ output [27, 28]. Their approach yields estimated marginal productivity differentials and wage differentials for workers with different characteristics, and a framework to test their equality.

Hellerstein and Neumark use Israeli labour market data to test whether the wage gap between men and women exceeds the gap between them (if any) in marginal productivity [27]. Hellerstein et al. use US population data to estimate wage and marginal productivity differentials for worker groups with different age, sex, and race characteristics [28]. Many recent studies have applied the Hellerstein et al. framework. For example, Haegeland and Klette analyze wage and productivity gaps among Norwegian workers grouped by sex, education and work experience [29]; van Ours and Stoeldraijer identify 13 studies on age, wage and productivity using linked employer-employee data [30].

Our theoretical framework is based on Pauly et al. [9]. They develop a general model to examine the magnitude and incidence of costs associated with absenteeism under alternative assumptions about firm size, the production function, the nature of the firm’s product, and the competitiveness of the labor market. We test two key theoretical predictions of their model using the Hellerstein et al. [27] and Hellerstein and Neumark [28] framework.

The first prediction is that the productivity loss associated with a worker’s absence will be larger than the wage in firms with team production. If a team worker is absent, the output of the entire team may be affected. Hence the impact on firm output exceeds the wage that would have been paid to the absent team worker. We test the hypothesis that the absence of team workers has a larger effect on firm-level production than wages (i.e., a significant difference between productivity effects and wage effects). In contrast, we hypothesize that the absence of non-team workers has a similar effect on production and wages.

The second prediction is that the difference between the wage and the productivity loss due to absence will be larger in small firms than large firms. While large firms can hire extra employees to ensure that a given output level can be maintained if a team worker is absent, small firms may not be able to afford this expense. We test whether the difference between productivity effects and wage effects is larger in small firms than large firms.

### Previous literature on the impact of absenteeism and team on wages and production

A related literature seeks to uncover factors that determine or affect worker absence by modeling absence [17, 31–35] or focuses on the association between health conditions and absenteeism [36–39]. Few studies have estimated the impact of absenteeism on wages or production, and none have examined whether their impact varies by team work status and firm size.

Allen estimates the trade-off between wages and expected absence via a hedonic wage equation using individual worker level data in 1970s, and the effect of absenteeism on output per man-hour via a plant-level production function for manufacturing [40]. He finds a small difference between the wage effect and the productivity effect. However, he uses different data for the effects and does not estimate the two equations simultaneously. Thus, the absence-rate coefficients from the two equations might not be comparable.

Several studies have estimated the impact of absenteeism on productivity using plant-level data. In the production function of Allen [40], the elasticity of the absence rate is −0.015, meaning an increase in the absence rate from 0.1 to 0.2 reduces the output per man-hour by 1%. In addition, Mefford examines the effect of unions on productivity in 31 plants of a large multinational firm from 1975 to 82 [41]. He also includes the absence rate into the production function and finds that the elasticity of the absence rate is −0.033, implying if the absence rate increases from 0.1 to 0.2, productivity will decrease by 2.3%. The direction of the estimated effect in our study is consistent with these previous studies yet the magnitude of the effect size is greater.

Coles et al. introduced the idea of the shadow cost of absenteeism: the relatively high wage paid by firms requiring a low level of absenteeism, to compensate workers for attending work reliably [17]. They use just-in-time as an indicator of an assembly line production process. Using individual worker level data, they find an association between higher wages and lower absence rates; however, the relationship is almost twice as steep in just-in-time firms contrasted to non-just-in-time firms.

### Measure of compensation

#### Wage rate versus the impact of absenteeism on aggregate wages

In the absenteeism literature, the measure of opportunity cost of absenteeism is usually proxied by the worker’s wage rate (wage per unit time) taken from firm data. In this paper, however, the wage cost of absenteeism comes from an estimate of the impact of worker absenteeism on aggregate wages for workers at a firm. It may differ from a direct measure of the wage rate because the equilibrium wage incorporates any effects of absenteeism as a compensating differential. For example, the observed wage per day may vary much less between a firm where (for some exogenous reasons) absenteeism is common and one where it is rare than does the estimate from our wage regression. Most importantly, with only an aggregate measure of output available, we prefer to use the aggregate wages at the firm level in order to obtain the most comparable estimates. As Hellerstein et al. pointed out, by jointly estimating the firm-level production function and wage equation, we can conduct straightforward statistical tests of the equality of wages and marginal productivity [27]. Furthermore, the biases from some unobservables are more likely to affect the estimated absenteeism impacts on productivity and wages similarly when both are estimated at the firm level. Their impact on the tests of the equality of marginal productivity and wages is therefore diminished.

#### Payroll and non-wage benefits

In our main analysis, we use payroll as a measure of compensation. Payroll or wage is only part of the total employee compensation. Non-wage benefits are also available to employees, e.g., health related benefits (e.g. dental care, life insurance), pay related benefits (e.g. severance allowances), or pension related benefits. As a robustness test, we also use the total compensation (payroll plus non-wage benefits) as the outcome in our wage equation.

### Measure of absenteeism

Because we are primarily interested in estimating the productivity loss due to illness for applications in health care economic evaluation studies, an ideal measure of absenteeism would reflect illness-related absences only. However, data limitations dictate that we rely on a broader measure of absenteeism. The WES data used in this study only measure absences due to paid sick leave, but not unpaid sick leave. Following the definition of Dionne and Dostie [32], our measure of absenteeism includes the number of days of paid sick leave; other paid leave encompassing education leave, disability leave, bereavement, marriage, jury duty, and union business; and unpaid leave. It does not include paid vacations, paid paternity/maternity leave, or absence due to strikes or lock-outs. Although our measure of absenteeism is broader than a pure measure of illness-related absenteeism, our findings are still useful to determine whether wages are a reasonable proxy of the productivity loss due to illness-related absenteeism under the assumption that illness-related absenteeism and other forms of paid and unpaid leave have a similar impact on wages and output.

## Methods

Our empirical analysis is based on two firm-level equations which we specify and estimate jointly: a production function and a wage equation. The production function is used to capture productivity effects related to absenteeism and team work at the firm level, and the wage equation is to capture the corresponding wage effects. By simultaneously estimating the two equations, we can compare the productivity effects with wage effects to determine the equality of marginal productivity and wages. The traditional approach of estimating the wage equation alone to measure the impact of absenteeism does not fully capture productivity differentials associated with different levels of absenteeism.

We think it is useful to baseline our results with an estimate of economy-wide aggregate effects. Thus we begin by estimating a baseline model that restricts the effect of absenteeism to be the same for team workers and non-team workers and in small and large firms. We subsequently relax these restrictions by assuming that absenteeism affects team workers and non-team workers differently, and then by estimating our model separately for small and large firms.

### Production function

Our baseline specification of the production function is an extension of the standard Cobb-Douglas [27, 28, 42, 43]. See Additional file 1: Appendix B for its complete deviation. Because the Cobb-Douglas form is restrictive, we assess the robustness of our estimates to more general alternatives described in Section 3.4.

For each workplace, we start with a simple Cobb-Douglas production function:

1 $$ \mathrm{In}\;{Q}_j=\alpha\;\mathrm{In}\;{L}_j^A+\beta\;\mathrm{In}\;{K}_j+\upeta {F}_j+{\mu}_j $$

where *Q*_*j*_ is output, measured as value added by firm $j,\ {L}_j^A$ is an aggregate labour input defined below, *K*_*j*_ is the capital stock, *F*_*j*_ is a matrix of various firm characteristics, *α, β* are the elasticity of output with respect to labour and capital, respectively, η is a vector of parameters for firm characteristics and *μ*_*j*_ is the error term.

We divide the labour input into different worker types, that is, workers with different characteristics such as age, sex, education, occupation and team participation. If the total number of characteristics is *I* and workers are divided into V_*i*_ categories by each characteristic *i*, then the total number of worker types will be ${\displaystyle {\prod}_{i=1}^I{V}_i}$. Our aggregate labour input ${L}_j^A$ can be simplified after making several assumptions: First, we assume perfect substitutability among all types of workers and different marginal productivity for each worker type [27, 28]. Second, we assume that the proportion or distribution of one type of worker defined by one characteristic is constant across all other characteristic groups, which is referred to as the *equi-proportionate restriction* [27, 28].^1^ Third, we assume the relative marginal productivity of two types of workers within one characteristic group is equal to those within another characteristic group, which is referred to as the *equal relative productivity restriction* [27, 28].^2^ Fourth, attendance rates have the same marginal impact on productivity for different worker types.

The aggregate labour input can then be written as (equation 8 from Additional file 1: Appendix B):

2 $$ {L}_j^A={\left(1-{a}_j\right)}^{\theta }{\lambda}_{0,I}{L}_j\left(1+\left({\gamma}_G-1\right){P}_{\;Gj}\right){\displaystyle \prod_{i=1}^{I-1}\left(1+{\displaystyle \sum_{v=1}^{Vi-1}\left({\gamma}_{iv}-1\right){P}_{ivj}}\right)} $$

where *a*_*j*_ is the absence rate in firm *j*, *L*_*j*_ is the number of all workers in the firm *j*, *P*_*Gj*_ is the proportion of team workers among all workers in the firm *j*, *i* = 1, 2, …, *I*-1 indicates worker characteristics other than team participation, *v*_*i*_ = 1, 2, …, *V*_*i*_-1 represents worker categories divided according to the worker characteristic *i*, ${P}_{ivj}=\frac{L_{ivj}}{L_j}$ is the proportion of the worker type *iv* among all workers in the firm *j*, *θ* is the parameter of (1-absence rate), i.e., the attendance impact on the marginal productivity for any worker type, *λ*_0,*I*_ is the marginal productivity for the reference group when work force is divided by *I* characteristics and absence rate = 0, *γ*_*G*_ is the relative marginal productivity of team workers compared to non-team workers, and ${\gamma}_{iv}=\frac{\lambda_{iv}}{\lambda_{io}}$ is the relative marginal productivity of one worker type *iv* to the worker type *i0* for each characteristic *i*.

By substituting ${L}_j^A$ into the simple production function, equation 1, we obtain our baseline specification (equations 9 and 10 from Additional file 1: Appendix B), i.e., a “restricted model” as follows:

3 $$ \begin{array}{l}\mathrm{In}\;{Q}_j={\beta}_0+\beta\;\mathrm{In}\;{K}_j+\alpha\;\mathrm{In}\;{L}_j+\alpha \theta\;\mathrm{In}\left(1-{\alpha}_j\right)+\alpha\;\mathrm{In}\left(1+\left({\gamma}_G-1\right){P}_{Gj}\right)\\ {}+\alpha {E}_j+\upeta Fj+\mu j\end{array} $$

Where

4 $$ {E}_j={\displaystyle \sum_{i=1}^{I-1}\mathrm{In}\left(1+{\displaystyle \sum_{v=1}^{Vi-1}\left({\gamma}_{iv}-1\right){P}_{ivj}}\right)} $$

*E*_*j*_ refers to workforce characteristics other than team participation, and *β*_0_ is a constant term that incorporates *a* In *λ*_0,*I*_.

In addition, we relax the fourth assumption for team-work participation, that is, the attendance impact on the marginal productivity for team workers (*θ*_*G*_) is different from that for non-team workers (*θ*_*N*_). A relatively “complete model” (equations 12 and 13 from Additional file 1: Appendix B) is therefore presented as:

5 $$ {L^A}_j={\lambda}_{0,I}{\left(1-{a}_j\right)}^{\theta_N}{L}_j\left(1+\left({\gamma}_G{\left(1-{a}_j\right)}^{\theta_G-{\theta}_N}-1\right){P}_{Gj}\right){\displaystyle \prod_{i=1}^{I-1}}\left(1+{\displaystyle \sum_{v=1}^{V_i-1}}\left({\gamma}_{iv}-1\right){P}_{ivj}\right) $$

and,

6 $$ \begin{array}{l} \ln {Q}_j={\beta}_0+\beta \ln {K}_j+\alpha \ln {L}_j\\ {}\kern1.68em +\alpha {\theta}_N \ln \left(1-{a}_j\right)+\alpha \ln \left(1+\left({\gamma}_G{\left(1-{a}_j\right)}^{\theta_G-{\theta}_N}-1\right){P}_{Gj}\right)\\ {}\kern1.68em +\upalpha {E}_j+\upeta {F}_j+{\mu}_j\end{array} $$

### Wage equation

Applying the same approach as above, wage effects can be estimated through the relationship between payroll and average absence rate and share of workers participating in a team at the firm level. We write the aggregate wage *w*_*j*_ as the sum of wage for each worker type. Applying the same assumptions in the production function, the aggregate wage can be simplified as:

7 $$ {w}_j={w}_{0,I}{\left(1-{a}_j\right)}^{\zeta }{L}_j\left(1+\left({\phi}_G-1\right){P}_{Gj}\right)\ {\displaystyle \prod_{i=1}^{I-1}}\left(1+{\displaystyle \sum_{v=1}^{V_i-1}}\left({\phi}_{iv}-1\right){P}_{ivj}\right) $$

where *w*_*j*_ is the annual payroll of firm *j*, *w*_0,*I*_ is the wage for the reference group when work force is divided by *I* characteristics, *ζ* is the parameter of attendance rate, i.e., the attendance impact on wages for any worker type, *ϕ*_*G*_ is the relative wage of team workers to non-team workers, ${\phi}_{iv}=\frac{w_{iv}}{w_{i0}}$ is the relative wage of one worker type *iv* to the worker type *i0* for each characteristic *i* other than team participation.

After log transforming equation 7, the “restricted model” for wage equation is written as:

8 $$ \ln {w}_j={\beta}_{w0}+{\beta}_w \ln {K}_j+{\alpha}_w \ln {L}_j+\zeta \ln \left(1-{a}_j\right)+ \ln \left(1+\left({\phi}_G-1\right){P}_{Gj}\right)+{E}_{wj}+{\upeta}_{\mathrm{w}}{F}_j+{\mu}_{w,j} $$

where,

9 $$ {E}_{wj}={\displaystyle \sum_{i=1}^{I-1}} \ln \left(1+{\displaystyle \sum_{v=1}^{V_i-1}}\left({\phi}_{iv}-1\right){P}_{ivj}\right) $$

*β*_*w*0_ is a constant term incorporating *w*_0,*I*_, *α*_*w*_, *β*_*w*_ are the elasticity of wage with respect to labour and capital, respectively, η_w_ is a vector of parameters for firm characteristics and *μ*_*w*,*j*_ is the error term.

Correspondingly, we assume the attendance impact on wages differ by team participation and thus the relatively “complete model” becomes:

10 $$ {w}_j={w}_{0,I}{\left(1-{a}_j\right)}^{\zeta_N}{L}_j\left(1+\left({\phi}_G{\left(1-{a}_j\right)}^{\zeta_G-{\zeta}_N}-1\right){P}_{Gj}\right){\displaystyle \prod_{i=1}^{I-1}}\left(1+{\displaystyle \sum_{v=1}^{V_i-1}}\left({\phi}_{iv}-1\right){P}_{ivj}\right) $$

and

11 $$ \begin{array}{l} \ln {w}_j={\beta}_{w0}+{\beta}_w \ln {K}_j+{\alpha}_w \ln {L}_j\\ {}\kern1.68em +{\zeta}_N \ln \left(1-{a}_j\right)+ \ln \left(1+\left({\phi}_G{\left(1-{a}_j\right)}^{\zeta_G-{\zeta}_N}-1\right){P}_{Gj}\right)\\ {}\kern1.68em +{E}_{wj}+{\upeta}_{\mathrm{w}}{F}_j+{\mu}_{w,j}\end{array} $$

where *ζ*_*N*_ is the impact of attendance rate for non-team workers and *ζ*_*G*_ is the impact of attendance rate for team workers.

### Estimation

We estimate the production function and wage equation simultaneously via nonlinear least squares (NLS) [27, 28]., under the assumption that errors are correlated across equations (nonlinear seemingly unrelated regression).^3^ All observations are weighted using linked weights provided by Statistics Canada. All standard errors are computed as Statistics Canada’s recommended procedure [44] using 100 sets of provided bootstrap sample weights.

Our null hypothesis of primary interest is that the attendance coefficient in the production function equals the coefficient in the wage equation. In the restricted model, the equality of marginal productivity and wage is tested by comparing the attendance coefficients, *θ* and *ζ*. In the complete model, we compare the two coefficients for team workers, *θ*_*G*_ and *ζ*_*G*_, and those for non-team workers, *θ*_*N*_ and *ζ*_*N*_, respectively. We also test the equality of relative productivity of team workers to non-team workers and their relative wage by comparing (*λ*_*G*_ − 1) and (*ϕ*_*G*_ − 1).

In order to examine whether parameter estimates vary by firm size, we conduct our analyses separately on two sub-samples: small firms with less than 20 employees and large firms (the remainder).

### Robustness

We undertake further analyses to assess the robustness of our estimates. First, we relax restrictions on the functional form of our production function by estimating a specification using the much more flexible translog form. Second, we re-estimate our model using total compensation (payroll plus non-wage benefits) instead of payroll as the outcome of the wage equation.

Third, a key issue in the estimation of production functions is the potential correlation between input levels and unobserved firm-specific productivity shocks. Firms that have a large positive productivity shock may respond by using more inputs, giving rise to an endogeneity issue [45]. Following Hellerstein et al. [27], we address this issue by using value-added as the measure of output in the production function to avoid estimating a coefficient on materials. We also attempt to correct for the potential bias by estimating the model on first differences, which eliminates the effect of any time-invariant unobserved heterogeneity that jointly affects productivity and wages. We also apply Levinsohn and Petrin’s approach [46] using intermediate inputs (expenses on materials which are subtracted out in our value-added production function) to address the simultaneity problem. Specifically, we estimate parameters of our value-added production function using NLS by adding a third-order or a fourth-order polynomial approximation in capital and material inputs [47].

Finally, we conduct sensitivity analyses to examine the impacts of some of the assumptions embodied in our baseline specification. We relax the equi-proportionate restriction between occupation, age, sex, education (> university bachelor versus bachelor and below) and team participation, respectively.^4^ That restriction also implies that the firm-average absence rate is common to all worker types. To test the impact of this assumption, we allow the average absence rate to differ for team workers and non-team workers in each firm. That is, the firm-average absence rate in the complete model is replaced with the firm-average absence rate of team workers and the absence rate of non-team workers, correspondingly, as follows.

12 $$ \begin{array}{l}{L^A}_j={\left(1-{a}_{Gj}\right)}^{\theta_G}{\lambda}_{G,0,I-1}{L}_{Gj}{\displaystyle \prod_{i=1}^{I-1}}\left(1+{\displaystyle \sum_{v=1}^{V_i-1}}\left({\gamma}_{iv}-1\right){P}_{ivj}\right)\\ {}\kern1.44em +{\left(1-{a}_{Nj}\right)}^{\theta_N}{\lambda}_{N,0,I-1}{L}_{Nj}{\displaystyle \prod_{i=1}^{I-1}}\left(1+{\displaystyle \sum_{v=1}^{V_i-1}}\left({\gamma}_{iv}-1\right){P}_{ivj}\right)\\ {}\kern1.56em ={\lambda}_{0,I}{\left(1-{a}_{Nj}\right)}^{\theta_N}{L}_j\\ {}\left(1+\left({\gamma}_G\frac{{\left(1-{a}_{Gj}\right)}^{\theta_G}}{{\left(1-{a}_{Nj}\right)}^{\theta_N}}-1\right){P}_{Gj}\right){\displaystyle \prod_{i=1}^{I-1}}\left(1+{\displaystyle \sum_{v=1}^{V_i-1}}\left({\gamma}_{iv}-1\right){P}_{ivj}\right)\end{array} $$

and

14 $$ \begin{array}{l} \ln {Q}_j={\beta}_0+\beta \ln {K}_j\\ {}\kern1.68em +\alpha \ln {L}_j+\alpha {\theta}_N \ln \left(1-{a}_{Nj}\right)+\alpha \ln \left(1+\left({\gamma}_G\frac{{\left(1-{a}_{Gj}\right)}^{\theta_G}}{{\left(1-{a}_{Nj}\right)}^{\theta_N}}-1\right){P}_{Gj}\right)\\ {}\kern1.68em +\upalpha {E}_j+\upeta {F}_j+{\mu}_j\end{array} $$

## Data

The WES is a survey of Canadian employers and employees conducted by Statistics Canada over the period 1999–2006 [48].^5^ These data have been used to estimate age-based wage and productivity differentials [49] and to compare wages and marginal productivity for workers with different levels of education and technology use [50, 51].

The sampling frame for the WES includes all Canadian workplaces^6^ in the Statistics Canada Business Registry that had paid employees in March of the survey year. The sampling frame for employees comprises all employees working at or on paid leave from the targeted workplaces in March. In each year between 1999 and 2006, Statistics Canada surveyed a representative sample of approximately 6000 workplaces. The initial sample of workplaces was refreshed in odd-number years (2001, 2003, and 2005) to reflect attrition and firm births. In 1999–2005, Statistics Canada randomly sampled approximately 20,000 employees of sampled firms. The number of employees sampled from a firm was proportional to size, up to a maximum of 24. In workplaces with fewer than 4 employees, all employees were sampled. Sampled workers were surveyed for two years, and a new sample of workers was drawn in the next odd-numbered year.

Ethical approval for this study is not required because it was based exclusively on the WES conducted by Statistics Canada and we did not directly approach the study subjects. Our analysis is based on the pooled data 1999, 2001, 2003, and 2005 cross-sections.^7^ We further restrict the sample to workplaces with at least one employee interviewed, operating for profit, and with positive output. Our sample includes 18,381 observations on 7766 unique workplaces. There are 7784 observations for small firms and 10,597 for large firms. Table 1 illustrates the transition from the gross workplace sample to our final sample in detail.

**Table 1:** Transition from the gross sample to the final sample

	Observations	Workplaces
Gross sample	43832	9372
At least one employee without attrition^a^	36579	8875
For profit	31786	7931
Value added >0	30416	7812
Odd years	18381	7766
Small firms	7784	3870
Large firms	10597	4385

^a^In even survey years, employees who had a different employer or left his employer and did not have a new employer were considered as attrition

### Outcome variables

Our outcome variable in the wage equation is the firm’s total annual payroll. Our outcomes variables in the production function is the firm’s output. Following Turcotte and Rennison [50, 51], we define output as value added, where value added is measured as annual gross operating revenues minus expenses on materials.^8^ Expenses on materials equal annual gross operating expenditures minus total gross payroll and expenditures on non-wage benefits and training.

### Independent variables of interest

Our measure of absenteeism is the absence rate of the firm’s employees. This is defined as the number of days of total leave taken by employees, including paid sick leave, other paid leave (e.g., education leave, disability leave, bereavement, marriage, jury duty, union business) and unpaid leave [32] in the past 12 months or since the employee started his/her current job (if less than 12 months), divided by the total number of ‘usual workdays’^9^ over the same time period. The absence rate for a firm is the average absence rate for the employees surveyed at that firm. We define the firm’s attendance rate as one minus the absence rate.

We identify workers as being a member of a team based on their reported participation in “a self-directed work group (semi-autonomous work group or mini-enterprise group) that has a high level of responsibility for a particular product or service area” [48].^10^ In our analysis, team workers are those who report participating in such a group ‘frequently’ or ‘always’ and non-team workers are those who report participating in such a group ‘occasionally’ or ‘never’.

The *L*_*j*_ in our baseline specification is measured by the number of total employees employed by each workplace. Estimation of our production function also requires a measure of the firm’s capital stock. Unfortunately, there is no such measure in the WES. We therefore impute the firm’s capital stock following the approach of Dostie [49] and Turcotte and Rennison [50, 51]. Our imputed capital measure equals the five-year average capital stock in the firm’s industry, divided by the number of firms in each industry represented by the WES. The industry capital stock measure is the geometric (infinite) end-year net stock of non-residential capital reported in CANSIM Table 031–0002, obtained from Statistics Canada.^11^

Control variables in our empirical specification include other characteristics of the firm’s workforce (firm-average proportion of employees grouped by age, sex, education, occupation, race, immigration status, and membership in union or collective bargaining agreement, separately, included in *E*_*j*_), workplace characteristics (an indicator for selling into an international market, an indicator for foreign country ownership, region, and industry included in *F*_*j*_), and calendar year dummies. More details on the definition of all variables we used in the study can be found in Additional file 1: Appendix A.

Table 2 provides descriptive statistics for variables used in our analysis. At the workplace level, the average absence rate is low (0.02), of which 65% is unpaid leave, 19% is paid sick leave and 16% is other paid leave. The share of workers in teamwork is 8%. The average age is 40 years old and the share of female workers is 54%. Only 38% of workplaces have at least 5 employees surveyed. The average number of employees per firm is 15 and most firms (85%) fall in the category of 1–19 employees. There are more large firms sampled in the WES survey than small firms (Table 1). However, the small firms are assigned higher sampling weights than large firms to represent their much greater number in the Canadian economy.

**Table 2:** Descriptive statistics at workplace level

Variables	Weighted mean	Standard deviation
Value added (,000)	1393.333	38.705
Log value added	12.526	0.026
Total wage (,000)	524.346	10.281
Log wage	11.892	0.021
Employment	14.982	0.242
Capital stock (,000)	1254.673	59.224
Absence rate	0.019	0.001
Proportion of workers participating in a team	0.079	0.003
Other workforce characteristics		
Age	40.472	0.175
Proportion of workers by age		
Age <35	0.353	0.006
35 ≤ Age < 55	0.525	0.007
55 ≤ Age	0.123	0.005
Proportion of female workers	0.542	0.007
Proportion of workers by level of education		
< High school	0.130	0.005
High school graduate only	0.203	0.007
Under university bachelor (completed/some college or university)	0.539	0.007
University bachelor	0.092	0.003
> University bachelor	0.035	0.002
Proportion of workers by occupation		
Managers/professionals	0.269	0.005
Technical/trades/marking/sales/clerical/administrative	0.463	0.007
Production workers	0.200	0.006
Others	0.068	0.004
Proportion of ethnic minorities	0.187	0.006
Proportion of immigrants	0.179	0.006
Proportion of employees with bargaining agreement	0.046	0.002
Workplace characteristics	%	
Establishment size		
1–19 employees	84.7	
20–99 employees	13.5	
100–499 employees	1.6	
500 employees or more	0.2	
Number of employees surveyed^a^		
1	12.3	
2	16.8	
3	22.9	
4	9.9	
> =5	38.0	
International market	5.1	
Foreign country owned	3.3	
Industry		
Forestry, mining, oil, and gas extraction	1.5	
Labour intensive tertiary manufacturing	3.3	
Primary product manufacturing	1.2	
Secondary product manufacturing	2.0	
Capital intensive tertiary manufacturing	2.6	
Construction	8.2	
Transportation, warehousing, wholesale	12.1	
Communication and other utilities	1.3	
Retail trade and consumer services	33.7	
Finance and insurance	5.3	
Real estate, rental and leasing operations	4.2	
Business services	13.2	
Education and health services	9.7	
Information and cultural industries	1.7	
Region		
Atlantic	8.3	
Quebec	21.0	
Ontario	37.2	
Alberta	11.7	
British Columbia	14.9	
Manitoba	3.0	
Saskatchewan	3.8	
Year^a^		
1999	25.2	
2001	24.2	
2003	24.2	
2005	26.3	

Employer weight is used for workplace characteristics; linked weight is used for workforce characteristics

^a^unweighted estimates

## Results

Table 3 presents parameter estimates for our baseline model, which provides an estimate of the economy-wide aggregate effect of absenteeism. With the full set of controls, our estimate of the overall effect of attendance on marginal productivity (0.46) is almost identical to its estimated effect on wages (0.47). We cannot reject the hypothesis that the two coefficients are the same at conventional significance levels. These coefficients can be interpreted as elasticities: a 1% decline in the attendance rate reduces productivity by 0.95*0.46% = 0.44%^12^ and wages by 0.47%.

**Table 3:** Parameter estimates for the restricted model

	Production	*P* value	Wage	*P* value
Baseline controls^a^				
Log (total no. of employees)	0.94 (0.02)***	<0.001	1.04 (0.01)***	<0.001
Log (capital)	0.04 (0.01)***	<0.001	0.05 (0.01)***	<0.001
Attendance rate	0.42 (0.12)***	<0.001	0.41 (0.07)***	<0.001
Team	0.66 (0.19)***	<0.001	0.40 (0.08)***	<0.001
Difference in attendance rate coefficients	0.01 (0.10)	0.958		
Difference in team coefficients	0.26 (0.14)*	0.056		
All controls^b^				
Log (total no. of employees)	0.95 (0.02)***	<0.001	1.08 (0.01)***	<0.001
Log (capital)	0.00 (0.01)	0.931	−0.03 (0.01)***	0.002
Attendance rate	0.46 (0.13)***	<0.001	0.47 (0.07)***	<0.001
Team	0.26 (0.11)**	0.021	0.08 (0.05)	0.110
Difference in attendance rate coefficients	−0.01 (0.10)	0.953		
Difference in team coefficients	0.18 (0.09)**	0.037		

^a^Model adjusted for employment, capital stock, and years; ^b^Adjusted for employment, capital stock, occupation, age, sex, education, race, immigrant, bargaining agreement, international market, foreign owned, region, industry and year; Standard error in the bracket; ^***^ *p* ≤ 0.01; ^**^0.01 < *p* ≤ 0.05; ^*^0.05 < *p* ≤ 0.1

**Fig. 1:**
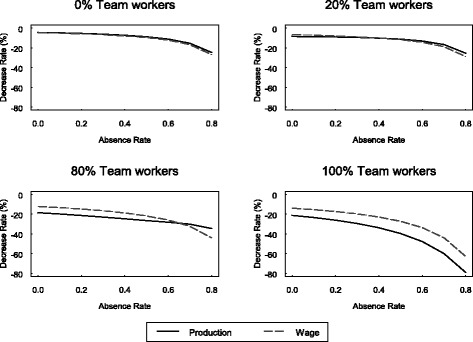
Rate at which output and wages decline for a 0.1 increase in the absence rate, at various levels of the firm’s absence rate and proportion of team workers

In Table 4, we relax our baseline specification by allowing the coefficient on the attendance rate to differ for team workers and non-team workers. The impact of attendance is much larger for team workers: coefficients are 2.38 in the production function and 1.43 in the wage equation. In this specification, the total effect of attendance (or absenteeism) on wages and productivity depends on both these coefficients and the proportion of employees that work in a team. Fig. 1. plots the rate at which productivity and wages decline when the absence rate increases by 0.1, at various levels of the firm’s absence rate and proportion of team workers. For example, at a firm where all employees work in teams, an increase in the absence rate from 0.1 to 0.2 reduces output by 23.4% and wages by 15.5%. At a firm where 20% of employees work in teams, output would only decline by 8.6% and wages by 7.2%. Correspondingly, the difference between the attendance impact on marginal productivity and the impact on wage for team workers is also larger than that for non-team workers (0.95 versus −0.02) (Table 4). However, the gap is not statistically significant.

**Table 4:** Parameter estimates for the complete model

	Production	*P* value	Wage	*P* value
Baseline controls^a^				
Log (total no. of employees)	0.94 (0.02)***	<0.001	1.04 (0.01)***	<0.001
Log (capital)	0.04 (0.01)***	<0.001	0.05 (0.01)***	<0.001
Attendance rate, non-team workers	0.37 (0.12)***	0.002	0.38 (0.07)***	<0.001
Attendance rate, team workers	2.78 (1.44)*	0.054	1.83 (0.84)**	0.029
Team	0.75 (0.17)***	<0.001	0.45 (0.08)***	<0.001
Difference in attendance coefficients, non-team workers	−0.01 (0.10)	0.876		
Difference in attendance coefficients, team workers	0.95 (0.95)	0.318		
Difference in team coefficients	0.30 (0.12)**	0.011		
All controls^b^				
Log (total no. of employees)	0.95 (0.02)***	<0.001	1.08 (0.01)***	<0.001
Log (capital)	0.00 (0.01)	0.935	−0.03 (0.01)***	0.002
Attendance rate, non-team workers	0.43 (0.13)***	<0.001	0.45 (0.07)***	<0.001
Attendance rate, team workers	2.38 (1.40)*	0.090	1.43 (0.75)*	0.058
Team	0.32 (0.12)**	0.012	0.10 (0.05)**	0.041
Difference in attendance coefficients, non-team workers	−0.02 (0.10)	0.816		
Difference in attendance coefficients, team workers	0.95 (1.00)	0.341		
Difference in team coefficients	0.21 (0.10)**	0.030		

^a^Model adjusted for employment, capital stock, and years

^b^Adjusted for employment, capital stock, occupation, age, sex, education, race, immigrant, bargaining agreement, international market, foreign owned, region, industry and year; Standard error in the bracket; ^***^ *p* ≤ 0.01; ^**^0.01 < *p* ≤ 0.05; ^*^0.05 < *p* ≤ 0.1

In Table 5, we further relax our baseline restrictions by estimating the model separately on sub-samples of small and large firms. The impact of non-team workers’ attendance on output and wages is smaller for small firms than for large firms: coefficients are 0.47 versus 1.32 in the production function and 0.44 versus 1.08 in the wage equation. As hypothesized, the difference between the two effects are not significantly different from zero in small firms (0.03) or large firms (0.24). In contrast, the impact of team workers’ attendance is much larger for small firms than for large firms. The productivity coefficients are 4.97 versus −0.76, and the wage coefficients are 2.25 versus −0.33, for small and large firms respectively. The difference between the attendance impact on output and that on wages is much larger in small firms (2.72) than in large firms (−0.43). The results suggest that in a large firm where all employees work in teams, absenteeism do not have any substantial impact on output or wages. On the other hand, absenteeism significantly reduces output and wages in small firms where all employees work in teams. The reduction in output is significantly higher than the reduction in wages at the 10% significance level. The results are consistent with our hypothesis that the absence of team workers has a larger effect on firm-level production than wages in small firms.

**Table 5:** Parameter estimates for the complete model by firm size

	Small firms				Large firms			
	Production	*P* value	Wage	*P* value	Production	*P* value	Wage	*P* value
Baseline controls^a^								
Log (total no. of employees)	0.87 (0.03)***	<0.001	1.04 (0.02)***	<0.001	1.07 (0.02)***	<0.001	1.01 (0.02)***	<0.001
Log (capital)	0.03 (0.01)***	0.005	0.04 (0.01)***	<0.001	0.09 (0.01)***	<0.001	0.08 (0.01)***	<0.001
Attendance rate, non-team workers	0.39 (0.14)***	0.005	0.36 (0.08)***	<0.001	1.95 (0.80)**	0.015	1.66 (0.58)***	0.004
Attendance rate, team workers	6.34 (2.25)***	0.005	3.01 (1.03)***	0.004	−0.57 (0.76)	0.449	−0.02 (0.70)	0.974
Team	0.75 (0.27)***	0.005	0.35 (0.10)***	<0.001	0.71 (0.15)***	<0.001	0.63 (0.12)***	<0.001
Difference in attendance coefficients, non-team workers	0.04 (0.11)	0.745			0.29 (0.36)	0.429		
Difference in attendance coefficients, team workers	3.33 (1.59)**	0.036			−0.55 (0.70)	0.431		
Difference in team coefficients	0.40 (0.21)*	0.056			0.08 (0.10)	0.433		
All controls^b^								
Log (total no. of employees)	0.88 (0.03)***	<0.001	1.07 (0.02)***	<0.001	1.10 (0.02)***	<0.001	1.03 (0.02)***	<0.001
Log (capital)	0.00 (0.02)	0.939	−0.03 (0.01)***	0.006	0.00 (0.01)	0.879	−0.01 (0.01)	0.263
Attendance rate, non-team workers	0.47 (0.14)***	0.001	0.44 (0.06)***	<0.001	1.32 (0.70)*	0.061	1.08 (0.47)**	0.021
Attendance rate, team workers	4.97 (1.87)***	0.008	2.25 (0.95)**	0.018	−0.76 (0.73)	0.300	−0.33 (0.64)	0.609
Team	0.33 (0.18)*	0.073	0.06 (0.06)	0.260	0.19 (0.10)*	0.054	0.09 (0.07)	0.213
Difference in attendance coefficients, non-team workers	0.03 (0.12)	0.811			0.24 (0.37)	0.511		
Difference in attendance coefficients, team workers	2.72 (1.49)*	0.068			−0.43 (0.72)	0.549		
Difference in team coefficients	0.27 (0.16)*	0.091			0.10 (0.07)	0.157		

Small firms are those with less than 20 employees; large firms are the remainder

^a^Model adjusted for employment, capital stock, and years

^b^Adjusted for employment, capital stock, occupation, age, sex, education, race, immigrant, bargaining agreement, international market, foreign owned, region, industry and year; Standard error in the bracket; ^***^ *p* ≤ 0.01; ^**^0.01 < *p* ≤ 0.05; ^*^0.05 < *p* ≤ 0.1

Our estimates of the relative productivity and the relative wage of team workers versus non-team workers imply that team workers are more productive and earn more than non-team workers in the full sample (Tables 3 and 4). This difference is statistically significant at the 5% level in the specification including all controls. The difference between relative productivity and relative wage is larger in small firms but smaller in large firms (Table 5). This implies that on average, the higher wages paid to team workers are considerably less than their productivity differential relative to non-team workers.

In Additional file 1: Appendix C, we present parameter estimates for all covariates that are included in the models of Table 3 to Table 5, as well as the results of various robustness checks. These include estimates based on a translog production function (estimated on the full sample) and using total compensation (payroll plus non-wage benefits) as the outcome of the wage equation. The estimates from these alternative specifications are similar to what we have obtained above. When we consider different absence rates for team workers and non-team workers, the coefficients do not change much, which suggests our main analyses are robust. When the equi-proportionate restriction is dropped for occupation, age, sex and education with team participation, the estimated coefficients change only slightly.^13^ Nevertheless, the qualitative nature of the results stay the same after relaxing these assumptions.

We have also re-estimated the model by excluding the capital stock and the attendance rate coefficients remain virtually identical. Therefore, we believe that our parameter estimates are robust to our (imperfect) measure of the capital stock.

We address the potential endogeneity of absenteeism and team work status in several ways. First, we have estimated the equations in first differences to remove any time invariant components of the model as a sensitivity analysis. The first differences estimates reported in Additional file 1: Appendix C are similar to the NLS estimates. Differencing does not eliminate the effect of correlated transitory shocks, however, and these are another potential source of bias. For example, a chemical spill accident may instigate sick leave and a reduction in output. Employee work attendance decisions also depend on the slope of the wage-absence tradeoff, which may introduced simultaneity problems [40]. In the presence of correlated transitory shocks or simultaneity, an instrumental variable (IV) approach [30, 52, 53] can be used to consistently estimate parameters. We have estimated IV specifications of our model using the lagged attendance rate as an instrument. However this instrument turns out to be weak (F-statistic < 10), and we were unable to identify other valid instruments in the WES. We therefore adopt the Levinsohn and Petrin approach [46] and obtain estimates similar to our main findings. Overall, we find our estimates to be stable across different specifications, and this provides strong evidence in support of our main conclusions that wages underestimate the productivity loss due to absenteeism in the presence of team production, especially in small firms.

## Discussion and conclusions

This study is the first to test the equality of the estimated absenteeism impacts on marginal productivity and wages using linked employer-employee data. Our findings support the theoretical predictions of Pauly et al. [9, 11] and provide compelling evidence that the productivity loss due to worker absence exceeds the wage for team workers, especially in small firms.

Our findings highlight that the productivity loss due to absenteeism among team workers substantially exceeds the wage in small firms. Interestingly, such a wage-productivity gap is absent in large firms. This may reflect differences in compensation policy between large and small firms, or differences in substitution possibilities. While team workers are more productive and earn higher wages than non-team workers, our findings further imply that their higher marginal productivity exceeds the wage premium they receive. Moreover, although we find that wages underestimate the productivity loss due to absenteeism for team workers, our estimates indicate that wages are reasonable estimate of the productivity loss due to absenteeism for non-team workers.

It is worth noticing that this study is an aggregate or ecologic study that has focused on the effect of team work at the firm level rather than at individual worker level due to a lack of individual-level output data. Thus, it might be subject to ecological bias. According to Greenland and Morgenstern [54], ecological bias can occur if confounders or other factors affecting output or wages are differentially distributed across firms (i.e., confounding by firms) or when the effects of absenteeism and team work on output and wages vary across firms (i.e., effect modification by firms). To minimize the bias, in our regression models, we have adjusted for firms’ workforce characteristics that potentially affect output and wages, which were derived from individual-level worker data. Furthermore, we are more interested in the equality of the effects of absenteeism and team work in the two equations: production equation and wage equation. By jointly estimating the two equations at the firm level, the bias is more likely to affect the estimated effects on output and wages similarly [27] and thus the impact of bias on the tests of the equality of marginal productivity and wages might be diminished.

Collectively, our findings help to value the burden of illness-related absenteeism, by establishing situations where the wage can be used as a reasonable proxy for lost productivity, and situations where it will underestimate the loss. This is important for economic evaluations that seek to measure the productivity gain or loss of a health care technology/intervention, which in turn can impact policy makers’ funding decisions. Other researchers have proposed a multiplier to adjust wages to estimate the productivity burden of illness or the productivity gain from a health care intervention [9, 11, 55]. Our study provides a justification for such a multiplier. In practice, the productivity loss can be estimated by calculating the measured number of absent workdays due to health problems, multiplied by the daily wage and the multiplier.

Finally, we have deliberately avoided being prescriptive with respect to the method that should be employed in measuring productivity losses in economic evaluations. We believe that the appropriate measurement approach (which we focus on above) has many dimensions and in this study our intention was to highlight the welfare economic implications of under/over estimating productivity impacts due to absenteeism. We hope that the debate on the inclusion or exclusion of productivity losses in economic evaluations will be informed by this work over and above the normative aspects of the controversy.

## Additional file

Additional file 1: Appendix A. Definition of variables. **Appendix B.** Equations. **Appendix C.** Additional results. (DOCX 89 kb)

## Footnotes

1 For example, older workers are assumed to be equally represented among team workers and non-team workers; the distribution of absence rate is the same across different worker types.

2 For instance, the relative marginal productivity of older workers versus younger workers among team workers is assumed to be the same as those among non-team workers.

3 We have also estimated the equations in first differences to remove the firm-level fixed effects. The estimates were similar to the NLS estimates but very imprecise due to the large number of implied firm effects relative to the sample size. The results are included in Appendix C.

4 For example, when dropping the restriction between sex and team participation, we allow the proportion of team workers to differ in female and male employees. The new specification includes the proportion of female team workers, proportion of male team workers and proportion of female non-team workers as the independent variables.

5 Only employers were surveyed in 2006.

6 Employers in Yukon, Nunavut, and the Northwest Territories are excluded from the survey, as are those operating in crop production, animal production, fishing, hunting and trapping, private households, religious organizations and public administration.

7 We do not use data from even-numbered years for two reasons. First, employee attrition is high in their second survey year and is likely nonrandom [56]. Second, many sampled workers change employers between survey years and only limited information is collected about their new employer.

8 Using value added as an output measure helps address the potential endogeneity of materials by avoiding estimation of a coefficient on materials [27, 50, 51]. Another advantage of a value-added specification is that it improves comparability of data across industries and across workplaces within industries when their degree of vertical integration differs [27].

9 The total number of usual workdays equals to the number of days per week that employees usually work multiplied by the number of weeks per year they usually work.

10 More information on self-directed work group was provided in the question, i.e., “In such systems, part of your pay is normally related to group performance. Self-directed work groups: 1) Are responsible for production of a fixed product or service, and have a high degree of autonomy in how they organize themselves to produce that product or service. 2) Act almost as ‘businesses within businesses’. 3) Often have incentives related to productivity, timeliness and quality. 4) While most have a designated leader, other members also contribute to the organization of the group’s activities.”

11 Although firms in the WES are classified into industries according to 6-digit North American Industry Classification System (NAICS) (a total of 837 unique industries), the capital stock information provided by Statistics Canada is only available for 247 industries at varying levels of NAICS detail (2–6 digits, depending on industry). The 247 industries are not exclusive because both higher level and lower level of their NACIS are included for some industries. Eventually, a total of exclusive 201 NACISs are used: 2 in 2 digits, 70 in 3 digits, 107 in 4 digits, 20 in 5 digits and 2 in 6 digits. Hence, to impute a net stock estimate, we had to impute some firm’s capital stock using the average value in a higher-level aggregate of the firm’s industry.

12 Note the output elasticity of labour is 0.95.

13 Results are not presented but will be available upon request.
