# Supplementary material for: Valuing productivity loss due to absenteeism: firm-level evidence from a Canadian linked employer-employee survey
Source: Health Econ Rev. 2017 Jan 19;7:3. doi: 10.1186/s13561-016-0138-y (PMC5247392; doi:10.1186/s13561-016-0138-y)
Supplement: Additional file 1: Appendix A. — Definition of variables. Appendix B. Equations. Appendix C. Additional results. (DOCX 89 kb) [file 13561_2016_138_MOESM1_ESM.docx]

Definition of variables

Outcome variables

**Value added**: Annual gross operating revenues minus expenses on materials.

**Expenses on material (proxy)**: Gross operating expenditures minus total gross payroll and expenses on non-wage benefits and on training.

**Wage**: Total gross payroll for all employees.

Independent variables of interest

**Absence rate:** The total number of days of leave taken by employees, including paid sick leave, other paid leave (e.g., education leave, disability leave, bereavement, marriage, jury duty, union business) and unpaid leave, divided by the total number of ‘usual workdays’ in the past twelve months/since the employee started his/her current job. The total number of usual workdays is equal to the number of days per week employees usually work multiplied by the number of weeks per year they usually work.

**Attendance rate:** 1 minus absence rate.

**Proportion of workers in team work (proxy):** Number of employees involved in team work divided by the number of employees surveyed in the workplace. The question asking about team work participation is “how frequently are you part of a self-directed work group (semi-autonomous work group or mini-enterprise group) that has a high level of responsibility for a particular product or service area? In such systems, part of your pay is normally related to group performance. Self-directed work groups: 1) Are responsible for production of a fixed product or service, and have a high degree of autonomy in how they organize themselves to produce that product or service. 2) Act almost as ‘businesses within businesses’. 3) Often have incentives related to productivity, timeliness and quality. 4) While most have a designated leader, other members also contribute to the organization of the group’s activities.” Workers are divided into two groups based on their participation frequency (‘frequently’ or ‘always’ (team work) versus ‘occasionally’ or ‘never’ (no team work) for participation in any team work).

Covariates

**Employment**: Number of people employed at each workplace.

**Capital stock (proxy):** The capital stock for a firm is approximated by using the average capital stock of the particular industry that the firm belongs to during the five years prior to the corresponding year. The industry capital stock corresponds to the geometric (infinite) end-year net stock of non-residential capital reported in Table 031-0002 of CANSIM from Statistics Canada (chained 2002 dollars). The firm capital stock is calculated by dividing the industry capital stock by the number of firms in each industry in the WES. The number of firms in each industry in the WES is calculated by adding the WES weight for each firm by industry.

**Proportion of workers by age (proxy):** Number of employees in an age group divided by the number of employees surveyed in the workplace. Three age groups were defined as 1) less than 35, 2) between 35 and 55, and 3) over 55.

**Proportion of female workers (proxy):** Number of female employees divided by the number of employees surveyed in the workplace.

**Proportion of workers by level of education (proxy):** Number of workers with a given education level divided by the number of employees surveyed in the workplace. We distinguish among the following levels of education: less than high school, high school graduate only, under university graduate (completed/some college or university below bachelor), University bachelor, and higher than a bachelor’s degree.

**Proportion of workers by occupation:** Number of workers in the given occupation group divided by the number of people employed in the workplace. Occupation types include 1) managers and professionals; 2) technical/trades, marketing/sales, and clerical/administrative; and 3) production workers with no trade/certification, operation and maintenance; 4) others.

**Proportion of employees with non-white ethnic minorities (proxy):** Number of employees from any ethnic, cultural and racial group other than Canadian, British, American, French, and other European groups divided by the number of employees surveyed in the workplace.

**Proportion of employees who were immigrants (proxy):** Number of employees born outside of Canada divided by the number of employees surveyed in the workplace.

**Proportion of employees with bargaining agreement (proxy):** Number of non-management employees with membership in a union or collective bargaining agreement divided by the number of employees in the workplace.

**International market:** Market with the highest market sales in percentage of total sales among “local”, “rest of Canada”, “USA or rest of the World”. Workplaces with an international market are those for which the most important market for sales is “USA or the rest of World”.

**Foreign country owned**: Workplaces where more than 50 percent of the assets of this workplace are held by foreign interest assets.

**Industry:** There are 14 industry categories: 1) Forestry, mining, oil, and gas extraction, 2) Labour intensive tertiary manufacturing, 3) Primary product manufacturing, 4) Secondary product manufacturing , 5) Capital intensive tertiary manufacturing, 6) Construction, 7) Transportation, warehousing, wholesale, 8) Communication and other utilities, 9) Retail trade and consumer services, 10) Finance and insurance, 11) Real estate, rental and leasing operations, 12) Business services, 13) Education and health services, 14) Information and cultural industries.

**Region:** There are seven categories: 1) Atlantic, 2) Quebec, 3) Ontario, 4) Alberta, 5) British Columbia, 6) Manitoba and 7) Saskatchewan.

Equations

According to the framework of Hellerstein et al. [1, 2], two firm-level equations are estimated jointly to measure and compare the relative productivity between different types of workers: a production function and a wage equation. A production function is used to capture the relative productivity between different types of workers and a wage equation is to capture the corresponding relative wage. The types of workers can be defined according to worker characteristics such as age, sex, occupations, education level, team work status, and health status. Workers are divided into different categories by their characteristics. A previous study has presented such a production function and wage equation to compare the relative productivity with the relative wage between types of workers [3]. In this study, we extend the two equations by incorporating absence rate to capture productivity effects related to absenteeism and team work at the firm level, and the corresponding wage effects.

Existing equations

Production function

Shown in a previous study [3], if we distinguish worker types according to I characteristics including team participation, age, sex, occupation, and education, the “restricted model” after making the *equi-proportionate restriction* and the *equal relative productivity restriction* [1–3] will be

|  | ${L^{A}}_{j}=\sum_{d=0}^{D_{I}-1} \lambda_{d}L_{dj}=\lambda_{0,I}L_{j}\prod_{i=1}^{I} \left( 1+\sum_{v=1}^{V_{i}-1} \left( \gamma_{iv}-1 \right)P_{ivj} \right)$ |  |
| --- | --- | --- |

and

|  | $\ln Q_{j}=\beta_{0}+\beta\ln K_{j}+\alpha\ln L_{j}+\alpha\sum_{i=1}^{I} \ln\left( 1+\sum_{v=1}^{V_{i}-1} \left( \gamma_{iv}-1 \right)P_{ivj} \right)+\eta F_{j}+\mu_{j}$ |  |
| --- | --- | --- |

where ${L^{A}}_{j}$ is an aggregate labour input, *D* is the total number of worker types, *L_d_* is the number of workers of type *d* in a firm *j*, *λ_d_* is the marginal productivity for workers of type *d*, $d=0$ refers to the reference group, $\lambda_{0,I}$ is the marginal productivity for the reference group when work force is divided by *I* characteristics, $L_{j}$ is the number of all workers in the firm *j*, *i* = 1, 2, …, *I* indicates the *i*th worker characteristics, *v_i_* = 1, 2, …, *V_i_*-1 represents worker categories divided according to the worker characteristic *i*, $\gamma_{iv}=\frac{\lambda_{iv}}{\lambda_{i0}}$ is the relative marginal productivity of one worker type *iv* to the worker type *i0* for each characteristic *i*, $P_{iv}=\frac{L_{iv}}{L}$ is the proportion of the worker type *iv* among all workers, $\beta_{0}$ is a constant term that incorporates $\alpha\ln\lambda_{0,I}$, $Q_{j}$ is output, measured as value added by firm *j*, $K_{j}$ is the capital stock, $F_{j}$ is a matrix of various firm characteristics, *α, β* are the elasticity of output with respect to labour and capital, respectively, $\eta$ is a vector of parameters for firm characteristics and $\mu_{j}$ is the error term [3].

Wage equation

Shown in our previous study [3], the aggregate wage is written as the sum of wage for each worker type

|  | $w=\sum_{d=0}^{D_{I}-1} w_{d}L_{d}$ |  |
| --- | --- | --- |

where $w_{d}$ is the wage for the worker type *d*.

Applying the same approach as in the case of marginal productivity, the “restricted model” for wage equation will be

|  | $w=w_{0,I}L \prod_{i=1}^{I} \left( 1+\sum_{v=1}^{V_{i}-1} \left( \phi_{iv}-1 \right)P_{iv} \right)$ |  |
| --- | --- | --- |

and

|  | $\ln w_{j}=\beta_{w0}+\beta_{w}\ln K_{j}+\alpha_{w}\ln L_{j}+\sum_{i=1}^{I} \ln\left( 1+\sum_{v=1}^{V_{i}-1} \left( \phi_{iv}-1 \right)P_{ivj} \right)+\eta_{w}F_{j}+\mu_{w,j}$ |  |
| --- | --- | --- |

where $w_{0,I}$ is the wage for the reference group when work force is divided by *I* characteristics, *i* stands for the number of characteristics, and *v* stands for the number worker categories divided according to the worker characteristic *i*, $\phi_{iv}=\frac{w_{iv}}{w_{i0}}$ is the relative wage of one worker type *iv* to the worker type *i0* for each characteristic *i*, $\beta_{w0}$ is a constant term that incorporates$\ln w_{0,I}$, $\alpha_{w}$*,* $\beta_{w}$ are the elasticity of wage with respect to labour and capital, respectively, $\eta_{w}$ is a vector of parameters for firm characteristics, $\mu_{w,j}$ is the error term.

Incorporating absence rate

Production function

We can incorporate absence rate into the aggregate function of productive labour in the following form:

|  | $L^{A}=\sum_{d=0}^{D_{I}-1} \lambda_{d}{\left( 1-a \right)^{\theta_{d}}L}_{d};0\leq a\leq1 and \theta_{d}>0;$ |  |
| --- | --- | --- |

where *a* is the absence rate in a firm based on the assumption that the absence rate is the same across different worker types, $\lambda_{d}\left( 1-a \right)^{\theta_{d}}$ is the marginal productivity for workers of type *d*, $\lambda_{d}$ is the marginal productivity for worker type *d* when absence rate = 0, and $\theta_{d}$ is the parameter of (1-absence rate), i.e., the attendance impact on the marginal productivity for workers of type *d*. This is a convenient form for estimation because it reduces to the standard model $L^{A}=\sum_{d=0}^{D_{I}-1} \lambda_{d}L_{d}$ if *a* = 0 (no absence) and $L^{A}=0$ if *a* = 1 (no attendance). Using this form, it can also be shown that the condition, $\frac{\partial L^{A}}{\partial a}<0$ , will hold.

If we assume that the attendance impacts on the marginal productivity for different worker type *d* are the same ($\theta_{d}=\theta$), then the productive labour function can be rewritten as

|  | $L^{A}=\left( 1-a \right)^{\theta}\sum_{d=0}^{D_{I}-1} \lambda_{d}L_{d}$ |  |
| --- | --- | --- |

where $\theta$ is the parameter of attendance rate for any worker type.

After making the *equi-proportionate restriction* and the *equal relative productivity restriction*, the “restricted model”, according to the equation (1), will be

|  | $L^{A}=\left( 1-a \right)^{\theta}\sum_{d=0}^{D_{I}-1} \lambda_{d}L_{d}=\left( 1-a \right)^{\theta}\lambda_{0,I}L \prod_{i=1}^{I} \left( 1+\sum_{v=1}^{V_{i}-1} \left( \gamma_{iv}-1 \right)P_{iv} \right)=\left( 1-a \right)^{\theta}\lambda_{0,I}L\left( 1+\left( \gamma_{G}-1 \right)P_{G} \right) \prod_{i=1}^{I-1} \left( 1+\sum_{v=1}^{V_{i}-1} \left( \gamma_{iv}-1 \right)P_{iv} \right)$ |  |
| --- | --- | --- |

and

|  | $\ln Q_{j}=\beta_{0}+\beta\ln K_{j}+\alpha\ln L_{j}+\alpha\theta\ln\left( 1-a_{j} \right)+\alpha\sum_{i=1}^{I} \ln\left( 1+\sum_{v=1}^{V_{i}-1} \left( \gamma_{iv}-1 \right)P_{ivj} \right)+\eta F_{j}+\mu_{j}=\beta_{0}+\beta\ln K_{j}+\alpha\ln L_{j}+\alpha\theta\ln\left( 1-a_{j} \right)+\alpha\ln\left( 1+\left( \gamma_{G}-1 \right)P_{Gj} \right)+\alpha E_{j}+\eta F_{j}+\mu_{j}$ |  |
| --- | --- | --- |

where

|  | $E_{j}=\sum_{i=1}^{I-1} \ln\left( 1+\sum_{v=1}^{V_{i}-1} \left( \gamma_{iv}-1 \right)P_{ivj} \right)$ |  |
| --- | --- | --- |

$\gamma_{G}$ is the relative marginal productivity of team workers to non-team workers, $P_{Gj}$ is the proportion of team workers among all workers at firm *j*, *i* = 1, 2, …, *I*-1, indicates characteristics other than team work participation.

If we assume that the attendance impact on the marginal productivity for team workers (G) is different from that for non-team workers (N), then the productive labour function (15) can be rewritten as

|  | $L^{A}=\left( 1-a \right)^{\theta_{G}}\sum_{d=0}^{\frac{D_{I}}{2}-1} \lambda_{Gd}L_{Gd}+\left( 1-a \right)^{\theta_{N}}\sum_{d=0}^{\frac{D_{I}}{2}-1} \lambda_{Nd}L_{Nd}$ |  |
| --- | --- | --- |

where $\theta_{G}$ is the parameter of attendance rate for team workers, $\theta_{N}$ is the parameter of attendance rate for non-team workers, $\lambda_{Gd}$ is the marginal productivity for the worker type *d* in team workers, $\lambda_{Nd}$ is the marginal productivity for the worker type *d* in non-team workers.

With the same restrictions as above, the relatively “complete model” will be

|  | $L^{A}=\left( 1-a \right)^{\theta_{G}}\lambda_{G,0,I-1}L_{G}\prod_{i=1}^{I-1} \left( 1+\sum_{v=1}^{V_{i}-1} \left( \gamma_{iv}-1 \right)P_{iv} \right)+\left( 1-a \right)^{\theta_{N}}\lambda_{N,0,I-1}L_{N}\prod_{i=1}^{I-1} \left( 1+\sum_{v=1}^{V_{i}-1} \left( \gamma_{iv}-1 \right)P_{iv} \right)=\lambda_{0,I}\left( 1-a \right)^{\theta_{N}}L\left( 1+\left( \gamma_{G}\left( 1-a \right)^{\theta_{G}-\theta_{N}}-1 \right)P_{G} \right)\prod_{i=1}^{I-1} \left( 1+\sum_{v=1}^{V_{i}-1} \left( \gamma_{iv}-1 \right)P_{iv} \right)$ |  |
| --- | --- | --- |

and

|  | $\ln Q_{j}=\beta_{0}+\beta\ln K_{j}+\alpha\ln L_{j}+\alpha\theta_{N}\ln\left( 1-a_{j} \right)+\alpha\ln\left( 1+\left( \gamma_{G}\left( 1-a_{j} \right)^{\theta_{G}-\theta_{N}}-1 \right)P_{Gj} \right)+\alpha E_{j}+\eta F_{j}+\mu_{j}$ |  |
| --- | --- | --- |

Wage equation

Correspondingly, we rewrite the aggregate wage as the sum of wage for each worker type incorporating into absence rate:

|  | $w=\sum_{d=0}^{D_{I}-1} w_{d}\left( 1-a \right)^{\zeta_{d}}L_{d}$ |  |
| --- | --- | --- |

where $w_{d}\left( 1-a \right)^{\zeta_{d}}$ is the wage for the worker type *d*, $w_{d}$ is the wage for the worker type *d* when absence=0, and $\zeta_{d}$ is the parameter of attendance rate, i.e., the attendance impact on wage for the worker type *d*.

If we assume that the attendance impacts on wage for different worker type *d* are the same ($\zeta_{d}=\zeta$) and make the two restrictions, then the “restricted model” will be

|  | $w=\left( 1-a \right)^{\zeta}\sum_{d=0}^{D_{I}-1} w_{d}L_{d}=w_{0,I}\left( 1-a \right)^{\zeta}L \prod_{i=1}^{I} \left( 1+\sum_{v=1}^{V_{i}-1} \left( \phi_{iv}-1 \right)P_{iv} \right)=w_{0,I}\left( 1-a \right)^{\zeta}L\left( 1+\left( \phi_{G}-1 \right)P_{G} \right)\prod_{i=1}^{I-1} \left( 1+\sum_{v=1}^{V_{i}-1} \left( \phi_{iv}-1 \right)P_{iv} \right)$ |  |
| --- | --- | --- |

and

|  | $\ln w_{j}=\beta_{w0}+\beta_{w}\ln K_{j}+\alpha_{w}\ln L_{j}+\zeta\ln\left( 1-a_{j} \right)+\ln\left( 1+\left( \phi_{G}-1 \right)P_{Gj} \right)+E_{wj}+\eta_{w}F_{j}+\mu_{w,j}$ |  |
| --- | --- | --- |

where

|  | $E_{wj}=\sum_{i=1}^{I-1} \ln\left( 1+\sum_{v=1}^{V_{i}-1} \left( \phi_{iv}-1 \right)P_{ivj} \right)$ |  |
| --- | --- | --- |

and $\phi_{G}$ is the relative wage of team workers to non-team workers.

If we assume the attendance impact on wage differs by team participation and make the two restrictions, the relatively “complete model” will be

|  | $w=\left( 1-a \right)^{\zeta_{G}}\sum_{d=0}^{\frac{D_{I}}{2}-1} w_{Gd}L_{Gd}+\left( 1-a \right)^{\zeta_{N}}\sum_{d=0}^{\frac{D_{I}}{2}-1} w_{Nd}L_{Nd}=w_{0,I}\left( 1-a \right)^{\zeta_{N}}L\left( 1+\left( \phi_{G}\left( 1-a \right)^{\zeta_{G}-\zeta_{N}}-1 \right)P_{G} \right)\prod_{i=1}^{I-1} \left( 1+\sum_{v=1}^{V_{i}-1} \left( \phi_{iv}-1 \right)P_{iv} \right)$ |  |
| --- | --- | --- |

and

|  | $\ln w_{j}=\beta_{w0}+\beta_{w}\ln K_{j}+\alpha_{w}\ln L_{j}+\zeta_{N}\ln\left( 1-a_{j} \right)+\ln\left( 1+\left( \phi_{G}\left( 1-a_{j} \right)^{\zeta_{G}-\zeta_{N}}-1 \right)P_{Gj} \right)+E_{wj}+\eta_{w}F_{j}+\mu_{w,j}$ |  |
| --- | --- | --- |

where $\zeta_{G}$ is the parameter of attendance rate for team workers, and $\zeta_{N}$ is the parameter of attendance rate for team workers.

Additional results

In the section C.1.1 and C.1.2, we present the nonlinear least squares (NLS) parameter estimates for all covariates that were included in the models for Tables 3-6 in the main manuscript. With NLS estimates, we are unable to determine whether a positive association between productivity and attendance is because lower-attendance workers sort into lower-productivity workplaces (with low-attendance and high-attendance workers being about equally productive), or because lower-attendance workers are less productive than high-attendance workers within workplaces.

Furthermore, the least squares estimates are likely to be biased. A potential source of bias is unobserved workplace-level heterogeneity that may be correlated with the quantities of labour inputs. For example, a consistently poor working environment or conditions may not only affect productivity and wages, but also worker health, leading to an increase in the number of sick days.

Therefore we also try to address these sources of unobserved heterogeneity by estimating the equations in first differences to remove workplace-level fixed effects using the same pooled odd-year data used for the NLS analysis [4–6]. The coefficient on attendance rate in this specification estimates the rate at which output declines within a firm when the firm’s attendance rate falls. The results show that the first difference estimates are similar to the NLS estimates but much less precisely estimated.

In the section C.2, we have also included the results from additional analyses using the translog production in the full sample. In the section C.3, we estimate the results using total compensation (payroll plus non-wage benefits) as the outcome of the wage equation. In the section C.4, we apply Levinsohn and Petrin’s approach [7] using intermediate inputs (expenses on materials which are subtracted out in our value-added production function) to solve this simultaneity problem. Specifically, we estimate parameters of our value-added production function using NLS by adding a third-order or a fourth-order polynomial approximation in capital and material inputs [8]. The sections C.5 and C.6 present the results when we consider different absence rates for team workers and non-team workers. In addition, we perform the first differences analysis using a different pooled data, the first differences between 1999 and 2000, between 2001 and 2002, and between 2003 and 2004 among the same workplaces with the same employees between the consecutive two years. Thus, those employees who were lost follow-up or left the current workplace or the workplaces lost follow-up are excluded from this analysis. The results are presented in the section C.7. Overall, the findings from these alternative specifications or estimation methods are similar to what we obtained in our main analyses, which suggests our main analyses are robust.

Baseline specification

Full sample: Cobb-Douglas production function

Table C.1. Parameter estimates using restricted models for all workplaces

|  | **NLS** | | | | **First differences** | | | |
| --- | --- | --- | --- | --- | --- | --- | --- | --- |
|  | **Production** | **Wage** | **Production** | **Wage** | **Production** | **Wage** | **Production** | **Wage** |
| Constant | 10.34 (0.12)*** | 9.41 (0.07)*** | 10.97 (0.21)*** | 10.46 (0.13)*** |  |  |  |  |
| Log (employment) | 0.94 (0.02)*** | 1.04 (0.01)*** | 0.95 (0.02)*** | 1.08 (0.01)*** | 0.60 (0.08)*** | 0.69 (0.04)*** | 0.61 (0.08)*** | 0.70 (0.04)*** |
| Log (stock) | 0.04 (0.01)*** | 0.05 (0.01)*** | 0.00 (0.01) | -0.03 (0.01)*** | -0.03 (0.03) | 0.00 (0.01) | -0.03 (0.03) | -0.01 (0.01) |
| Attendance rate | 0.42 (0.12)*** | 0.41 (0.07)*** | 0.46 (0.13)*** | 0.47 (0.07)*** | 0.42 (0.40) | 0.05 (0.11) | 0.44 (0.38) | 0.05 (0.11) |
| Team | 0.66 (0.19)*** | 0.40 (0.08)*** | 0.26 (0.11)** | 0.08 (0.05) | 0.08 (0.14) | -0.01 (0.04) | 0.08 (0.15) | -0.01 (0.04) |
| 35 ≤ Age < 55 |  |  | 0.28 (0.08)*** | 0.26 (0.04)*** |  |  | 0.01 (0.09) | 0.05 (0.03)* |
| 55≤ Age |  |  | -0.01 (0.17) | 0.18 (0.06)*** |  |  | -0.06 (0.23) | 0.00 (0.04) |
| Female |  |  | -0.24 (0.06)*** | -0.25 (0.02)*** |  |  | 0.08 (0.18) | -0.02 (0.04) |
| > a bachelor’s degree |  |  | 0.26 (0.12)** | 0.32 (0.08)*** |  |  | -0.20 (0.12)* | 0.05 (0.06) |
| Managers/professionals |  |  | 0.61 (0.15)*** | 0.80 (0.11)*** |  |  | 0.20 (0.24) | 0.09 (0.07) |
| Technical/sales/clerical |  |  | 0.69 (0.13)*** | 0.42 (0.07)*** |  |  | 0.08 (0.14) | 0.02 (0.04) |
| Others |  |  | -0.14 (0.09) | -0.05 (0.06) |  |  | -0.01 (0.23) | 0.08 (0.06) |
| Minorities |  |  | 0.00 (0.08) | -0.05 (0.04) |  |  | 0.05 (0.16) | -0.02 (0.03) |
| Immigrants |  |  | -0.03 (0.08) | -0.06 (0.04) |  |  | 0.17 (0.24) | 0.00 (0.04) |
| Bargaining agreement |  |  | 0.32 (0.14)** | 0.16 (0.05)*** |  |  | 0.08 (0.34) | -0.02 (0.06) |
| International market |  |  | 0.37 (0.08)*** | 0.12 (0.03)*** |  |  |  |  |
| Foreign owned |  |  | 0.57 (0.09)*** | 0.22 (0.04)*** |  |  |  |  |
| Labour tertiary manufacturing |  |  | -0.44 (0.07)*** | -0.32 (0.04)*** |  |  |  |  |
| Primary product manufacturing |  |  | -0.28 (0.07)*** | -0.17 (0.04)*** |  |  |  |  |
| Secondary product manufacturing |  |  | -0.24 (0.07)*** | -0.12 (0.04)*** |  |  |  |  |
| Capital tertiary manufacturing |  |  | -0.27 (0.09)*** | -0.15 (0.04)*** |  |  |  |  |
| Construction |  |  | -0.42 (0.12)*** | -0.18 (0.05)*** |  |  |  |  |
| Transportation |  |  | -0.13 (0.07)* | -0.14 (0.04)*** |  |  |  |  |
| Communication |  |  | -0.42 (0.07)*** | -0.24 (0.04)*** |  |  |  |  |
| Retail trade |  |  | -0.73 (0.09)*** | -0.79 (0.05)*** |  |  |  |  |
| Finance |  |  | -0.02 (0.09) | -0.12 (0.05)*** |  |  |  |  |
| Real estate |  |  | -0.43 (0.11)*** | -0.45 (0.05)*** |  |  |  |  |
| Business services |  |  | -0.34 (0.09)*** | -0.32 (0.05)*** |  |  |  |  |
| Education |  |  | -0.24 (0.10)** | -0.46 (0.06)*** |  |  |  |  |
| Information |  |  | -0.58 (0.11)*** | -0.31 (0.05)*** |  |  |  |  |
| Atlantic |  |  | -0.14 (0.10) | -0.15 (0.04)*** |  |  |  |  |
| Quebec |  |  | -0.13 (0.06)** | -0.19 (0.03)*** |  |  |  |  |
| Alberta |  |  | -0.07 (0.06) | -0.06 (0.03)** |  |  |  |  |
| British Columbia |  |  | -0.13 (0.06)** | -0.03 (0.03) |  |  |  |  |
| Manitoba |  |  | -0.04 (0.09) | -0.17 (0.05)*** |  |  |  |  |
| Saskatchewan |  |  | -0.27 (0.09)*** | -0.22 (0.04)*** |  |  |  |  |
| Year01 | -0.11 (0.06)* | -0.03 (0.03) | -0.13 (0.05)** | -0.07 (0.03)** |  |  |  |  |
| Year03 | -0.20 (0.07)*** | -0.08 (0.04)** | -0.19 (0.07)*** | -0.07 (0.03)** | 0.02 (0.04) | 0.04 (0.02)** | 0.02 (0.04) | 0.04 (0.02)** |
| Year05 | -0.12 (0.06)** | -0.05 (0.03)* | -0.10 (0.06)* | -0.04 (0.03) | 0.07 (0.03)** | 0.05 (0.02)*** | 0.07 (0.03)** | 0.05 (0.02)*** |
| Difference in attendance rate coefficients | 0.01 (0.10) |  | -0.01 (0.10) |  | 0.37 (0.36) |  | 0.38 (0.34) |  |
| Difference in team coefficients | 0.26 (0.14)* |  | 0.18 (0.09)** |  | 0.09 (0.13) |  | 0.09 (0.14) |  |

Odd-year data are used; †Model adjusted for employment, capital stock, and years; ‡ Nonlinear Least Squares (NLS) estimates adjusted for employment, capital stock, occupation, age, sex, education, race, immigrant, bargaining agreement, international market, foreign owned, region, industry and year; First differences estimates adjusted for employment, capital stock, occupation, age, sex, education, race, immigrant, bargaining agreement, and year; Standard error in the bracket; ^***^p≤0.01; ^**^0.01<p≤0.05; ^*^0.05<p≤0.1

Table C.2. Parameter estimates using complete models for all workplaces

|  | **NLS** | | | | **First differences** | | | |
| --- | --- | --- | --- | --- | --- | --- | --- | --- |
|  | **Production** | **Wage** | **Production** | **Wage** | **Production** | **Wage** | **Production** | **Wage** |
| Constant | 10.34 (0.12)*** | 9.41 (0.07)*** | 10.97 (0.21)*** | 10.46 (0.13)*** |  |  |  |  |
| Log (employment) | 0.94 (0.02)*** | 1.04 (0.01)*** | 0.95 (0.02)*** | 1.08 (0.01)*** | 0.60 (0.08)*** | 0.69 (0.04)*** | 0.61 (0.08)*** | 0.70 (0.04)*** |
| Log (stock) | 0.04 (0.01)*** | 0.05 (0.01)*** | 0.00 (0.01) | -0.03 (0.01)*** | -0.03 (0.03) | 0.00 (0.01) | -0.03 (0.03) | -0.01 (0.01) |
| Attendance rate, non-team workers | 0.37 (0.12)*** | 0.38 (0.07)*** | 0.43 (0.13)*** | 0.45 (0.07)*** | 0.27 (0.39) | 0.01 (0.11) | 0.29 (0.37) | 0.01 (0.11) |
| Attendance rate, team workers | 2.78 (1.44)* | 1.83 (0.84)** | 2.38 (1.40)* | 1.43 (0.75)* | 2.72 (2.08) | 0.71 (0.62) | 2.73 (1.92) | 0.71 (0.64) |
| Team | 0.75 (0.17)*** | 0.45 (0.08)*** | 0.32 (0.12)** | 0.10 (0.05)** | 0.13 (0.17) | 0.00 (0.04) | 0.14 (0.18) | 0.00 (0.04) |
| 35 ≤ Age < 55 |  |  | 0.28 (0.08)*** | 0.26 (0.04)*** |  |  | 0.01 (0.09) | 0.05 (0.03)* |
| 55≤ Age |  |  | -0.01 (0.17) | 0.18 (0.06)*** |  |  | -0.06 (0.23) | 0.00 (0.04) |
| Female |  |  | -0.24 (0.06)*** | -0.25 (0.02)*** |  |  | 0.08 (0.17) | -0.02 (0.04) |
| > a bachelor’s degree |  |  | 0.26 (0.12)** | 0.32 (0.08)*** |  |  | -0.20 (0.12)* | 0.05 (0.06) |
| Managers/professionals |  |  | 0.61 (0.15)*** | 0.80 (0.11)*** |  |  | 0.21 (0.24) | 0.10 (0.07) |
| Technical/sales/clerical |  |  | 0.69 (0.13)*** | 0.42 (0.07)*** |  |  | 0.08 (0.15) | 0.02 (0.04) |
| Others |  |  | -0.14 (0.09) | -0.05 (0.06) |  |  | -0.02 (0.23) | 0.08 (0.06) |
| Minorities |  |  | 0.00 (0.08) | -0.05 (0.04) |  |  | 0.05 (0.15) | -0.02 (0.03) |
| Immigrants |  |  | -0.03 (0.08) | -0.06 (0.04) |  |  | 0.16 (0.24) | 0.00 (0.04) |
| Bargaining agreement |  |  | 0.33 (0.14)** | 0.16 (0.05)*** |  |  | 0.08 (0.34) | -0.02 (0.06) |
| International market |  |  | 0.37 (0.08)*** | 0.12 (0.03)*** |  |  |  |  |
| Foreign owned |  |  | 0.57 (0.09)*** | 0.22 (0.04)*** |  |  |  |  |
| Labour tertiary manufacturing |  |  | -0.45 (0.07)*** | -0.32 (0.04)*** |  |  |  |  |
| Primary product manufacturing |  |  | -0.29 (0.07)*** | -0.17 (0.04)*** |  |  |  |  |
| Secondary product manufacturing |  |  | -0.24 (0.07)*** | -0.13 (0.04)*** |  |  |  |  |
| Capital tertiary manufacturing |  |  | -0.28 (0.09)*** | -0.15 (0.04)*** |  |  |  |  |
| Construction |  |  | -0.43 (0.12)*** | -0.18 (0.05)*** |  |  |  |  |
| Transportation |  |  | -0.14 (0.07)* | -0.14 (0.04)*** |  |  |  |  |
| Communication |  |  | -0.43 (0.07)*** | -0.25 (0.04)*** |  |  |  |  |
| Retail trade |  |  | -0.74 (0.09)*** | -0.80 (0.05)*** |  |  |  |  |
| Finance |  |  | -0.02 (0.09) | -0.12 (0.05)*** |  |  |  |  |
| Real estate |  |  | -0.43 (0.11)*** | -0.45 (0.05)*** |  |  |  |  |
| Business services |  |  | -0.35 (0.09)*** | -0.32 (0.05)*** |  |  |  |  |
| Education |  |  | -0.24 (0.10)** | -0.46 (0.06)*** |  |  |  |  |
| Information |  |  | -0.58 (0.11)*** | -0.31 (0.05)*** |  |  |  |  |
| Atlantic |  |  | -0.14 (0.10) | -0.15 (0.04)*** |  |  |  |  |
| Quebec |  |  | -0.14 (0.06)** | -0.19 (0.03)*** |  |  |  |  |
| Alberta |  |  | -0.07 (0.06) | -0.06 (0.03)** |  |  |  |  |
| British Columbia |  |  | -0.12 (0.06)** | -0.03 (0.03) |  |  |  |  |
| Manitoba |  |  | -0.04 (0.09) | -0.17 (0.05)*** |  |  |  |  |
| Saskatchewan |  |  | -0.27 (0.09)*** | -0.22 (0.04)*** |  |  |  |  |
| Year01 | -0.11 (0.06)* | -0.03 (0.03) | -0.13 (0.05)** | -0.07 (0.03)** |  |  |  |  |
| Year03 | -0.20 (0.07)*** | -0.08 (0.04)** | -0.19 (0.07)*** | -0.06 (0.03)** | 0.02 (0.04) | 0.04 (0.02)** | 0.02 (0.04) | 0.04 (0.02)** |
| Year05 | -0.11 (0.06)** | -0.05 (0.03)* | -0.10 (0.06)* | -0.04 (0.03) | 0.07 (0.03)** | 0.05 (0.02)*** | 0.07 (0.03)** | 0.06 (0.02)*** |
| Difference in attendance coefficients, non-team workers | -0.01 (0.10) |  | -0.02 (0.10) |  | 0.27 (0.35) |  | 0.28 (0.33) |  |
| Difference in attendance coefficients, team workers | 0.95 (0.95) |  | 0.95 (1.00) |  | 2.02 (1.85) |  | 2.02 (1.70) |  |
| Difference in team coefficients | 0.30 (0.12)** |  | 0.21 (0.10)** |  | 0.13 (0.16) |  | 0.14 (0.17) |  |

Sub-samples: Cobb-Douglas production function

Table C.3. Parameter estimates using complete models for small firms and large firms

|  | **NLS (small firms)** | | | | **NLS (large firms)** | | | |
| --- | --- | --- | --- | --- | --- | --- | --- | --- |
|  | **Production** | **Wage** | **Production** | **Wage** | **Production** | **Wage** | **Production** | **Wage** |
| Constant | 10.56 (0.15)*** | 9.51 (0.09)*** | 11.09 (0.27)*** | 10.56 (0.17)*** | 9.36 (0.19)*** | 8.93 (0.14)*** | 10.36 (0.25)*** | 10.25 (0.17)*** |
| Log (employment) | 0.87 (0.03)*** | 1.04 (0.02)*** | 0.88 (0.03)*** | 1.07 (0.02)*** | 1.07 (0.02)*** | 1.01 (0.02)*** | 1.10 (0.02)*** | 1.03 (0.02)*** |
| Log (stock) | 0.03 (0.01)*** | 0.04 (0.01)*** | 0.00 (0.02) | -0.03 (0.01)*** | 0.09 (0.01)*** | 0.08 (0.01)*** | 0.00 (0.01) | -0.01 (0.01) |
| Attendance rate, non-team workers | 0.39 (0.14)*** | 0.36 (0.08)*** | 0.47 (0.14)*** | 0.44 (0.06)*** | 1.95 (0.80)** | 1.66 (0.58)*** | 1.32 (0.70)* | 1.08 (0.47)** |
| Attendance rate, team workers | 6.34 (2.25)*** | 3.01 (1.03)*** | 4.97 (1.87)*** | 2.25 (0.95)** | -0.57 (0.76) | -0.02 (0.70) | -0.76 (0.73) | -0.33 (0.64) |
| Team | 0.75 (0.27)*** | 0.35 (0.10)*** | 0.33 (0.18)* | 0.06 (0.06) | 0.71 (0.15)*** | 0.63 (0.12)*** | 0.19 (0.10)* | 0.09 (0.07) |
| 35 ≤ Age < 55 |  |  | 0.28 (0.09)*** | 0.24 (0.04)*** |  |  | 0.45 (0.11)*** | 0.40 (0.09)*** |
| 55≤ Age |  |  | -0.06 (0.18) | 0.16 (0.06)*** |  |  | 0.44 (0.17)*** | 0.40 (0.10)*** |
| Female |  |  | -0.25 (0.07)*** | -0.24 (0.03)*** |  |  | -0.27 (0.05)*** | -0.33 (0.04)*** |
| > a bachelor’s degree |  |  | 0.25 (0.14)* | 0.30 (0.08)*** |  |  | 0.16 (0.18) | 0.46 (0.13)*** |
| Managers/professionals |  |  | 0.62 (0.18)*** | 0.75 (0.12)*** |  |  | 1.32 (0.26)*** | 1.55 (0.22)*** |
| Technical/sales/clerical |  |  | 0.67 (0.17)*** | 0.37 (0.08)*** |  |  | 0.97 (0.13)*** | 0.66 (0.10)*** |
| Others |  |  | -0.15 (0.11) | -0.06 (0.07) |  |  | -0.17 (0.08)** | -0.05 (0.08) |
| Minorities |  |  | -0.01 (0.09) | -0.05 (0.05) |  |  | 0.06 (0.09) | -0.03 (0.05) |
| Immigrants |  |  | -0.04 (0.09) | -0.08 (0.04)* |  |  | -0.11 (0.09) | 0.08 (0.07) |
| Bargaining agreement |  |  | 0.45 (0.29) | 0.19 (0.10)** |  |  | 0.06 (0.06) | 0.12 (0.04)*** |
| International market |  |  | 0.47 (0.11)*** | 0.17 (0.03)*** |  |  | -0.04 (0.05) | -0.02 (0.06) |
| Foreign owned |  |  | 0.74 (0.13)*** | 0.32 (0.06)*** |  |  | 0.20 (0.06)*** | 0.03 (0.06) |
| Labour tertiary manufacturing |  |  | -0.52 (0.08)*** | -0.35 (0.05)*** |  |  | -0.15 (0.12) | -0.28 (0.05)*** |
| Primary product manufacturing |  |  | -0.41 (0.08)*** | -0.25 (0.05)*** |  |  | -0.06 (0.11) | -0.09 (0.05)* |
| Secondary product manufacturing |  |  | -0.22 (0.09)** | -0.09 (0.05)* |  |  | -0.14 (0.11) | -0.21 (0.06)*** |
| Capital tertiary manufacturing |  |  | -0.29 (0.10)*** | -0.16 (0.05)*** |  |  | -0.14 (0.11) | -0.17 (0.05)*** |
| Construction |  |  | -0.43 (0.14)*** | -0.18 (0.05)*** |  |  | -0.29 (0.10)*** | -0.23 (0.06)*** |
| Transportation |  |  | -0.12 (0.09) | -0.13 (0.05)*** |  |  | -0.13 (0.11) | -0.22 (0.06)*** |
| Communication |  |  | -0.49 (0.08)*** | -0.26 (0.05)*** |  |  | -0.31 (0.11)*** | -0.31 (0.05)*** |
| Retail trade |  |  | -0.73 (0.10)*** | -0.79 (0.06)*** |  |  | -0.67 (0.10)*** | -0.82 (0.06)*** |
| Finance |  |  | -0.03 (0.11) | -0.14 (0.05)** |  |  | -0.01 (0.12) | -0.17 (0.06)*** |
| Real estate |  |  | -0.44 (0.12)*** | -0.45 (0.05)*** |  |  | -0.13 (0.13) | -0.20 (0.09)** |
| Business services |  |  | -0.35 (0.11)*** | -0.33 (0.06)*** |  |  | -0.36 (0.13)*** | -0.35 (0.06)*** |
| Education |  |  | -0.21 (0.12)* | -0.46 (0.07)*** |  |  | -0.65 (0.12)*** | -0.66 (0.07)*** |
| Information |  |  | -0.62 (0.14)*** | -0.30 (0.06)*** |  |  | -0.47 (0.12)*** | -0.36 (0.07)*** |
| Atlantic |  |  | -0.17 (0.11) | -0.16 (0.05)*** |  |  | 0.01 (0.09) | -0.12 (0.05)** |
| Quebec |  |  | -0.16 (0.07)** | -0.20 (0.03)*** |  |  | -0.12 (0.07)* | -0.15 (0.05)*** |
| Alberta |  |  | -0.08 (0.07) | -0.06 (0.04) |  |  | -0.05 (0.06) | -0.08 (0.04)* |
| British Columbia |  |  | -0.14 (0.06)** | -0.03 (0.03) |  |  | -0.04 (0.07) | -0.01 (0.03) |
| Manitoba |  |  | -0.06 (0.10) | -0.17 (0.06)*** |  |  | 0.03 (0.11) | -0.17 (0.04)*** |
| Saskatchewan |  |  | -0.31 (0.10)*** | -0.25 (0.05)*** |  |  | -0.01 (0.09) | 0.00 (0.07) |
| Year01 | -0.09 (0.06) | -0.04 (0.03) | -0.11 (0.06)* | -0.07 (0.03)** | -0.18 (0.07)** | 0.05 (0.04) | -0.24 (0.06)*** | -0.01 (0.03) |
| Year03 | -0.20 (0.08)** | -0.11 (0.05)** | -0.19 (0.08)** | -0.08 (0.03)** | -0.16 (0.07)** | 0.13 (0.05)*** | -0.24 (0.06)*** | 0.05 (0.04) |
| Year05 | -0.10 (0.06) | -0.07 (0.03)** | -0.08 (0.07) | -0.05 (0.03) | -0.17 (0.07)** | 0.09 (0.04)** | -0.26 (0.06)*** | 0.01 (0.04) |
| Difference in attendance coefficients, non-team workers | 0.04 (0.11) |  | 0.03 (0.12) |  | 0.29 (0.36) |  | 0.24 (0.37) |  |
| Difference in attendance coefficients, team workers | 3.33 (1.59)** |  | 2.72 (1.49)* |  | -0.55 (0.70) |  | -0.43 (0.72) |  |
| Difference in team coefficients | 0.40 (0.21)* |  | 0.27 (0.16)* |  | 0.08 (0.10) |  | 0.10 (0.07) |  |

Full sample: Translog production function

Table C.4. Parameter estimates using restricted models

|  | **NLS** | | | | **First differences** | | | |
| --- | --- | --- | --- | --- | --- | --- | --- | --- |
|  | **Production** | **Wage** | **Production** | **Wage** | **Production** | **Wage** | **Production** | **Wage** |
| Constant | 13.13 (0.53)*** | 9.41 (0.07)*** | 13.52 (0.82)*** | 10.46 (0.13)*** |  |  |  |  |
| Log (employment) | 0.74 (0.07)*** | 1.04 (0.01)*** | 0.79 (0.08)*** | 1.08 (0.01)*** | 0.87 (0.28)*** | 0.69 (0.04)*** | 0.90 (0.29)*** | 0.70 (0.04)*** |
| Log (stock) | -0.38 (0.09)*** | 0.05 (0.01)*** | -0.38 (0.12)*** | -0.03 (0.01)*** | -0.24 (0.24) | 0.00 (0.01) | -0.24 (0.24) | -0.01 (0.01) |
| Attendance rate | 0.61 (0.18)*** | 0.42 (0.07)*** | 0.60 (0.18)*** | 0.47 (0.07)*** | 0.45 (0.44) | 0.05 (0.11) | 0.47 (0.42) | 0.05 (0.11) |
| Team | 0.73 (0.23)*** | 0.40 (0.08)*** | 0.25 (0.14)* | 0.08 (0.05) | 0.03 (0.09) | -0.01 (0.04) | 0.03 (0.09) | -0.01 (0.04) |
| 35 ≤ Age < 55 |  |  | 0.33 (0.11)*** | 0.26 (0.04)*** |  |  | 0.01 (0.06) | 0.05 (0.03)* |
| 55≤ Age |  |  | -0.04 (0.22) | 0.18 (0.06)*** |  |  | -0.03 (0.15) | 0.00 (0.04) |
| Female |  |  | -0.30 (0.07)*** | -0.25 (0.02)*** |  |  | 0.04 (0.11) | -0.02 (0.04) |
| > a bachelor’s degree |  |  | 0.29 (0.15)* | 0.32 (0.08)*** |  |  | -0.16 (0.09)* | 0.05 (0.06) |
| Managers/professionals |  |  | 0.81 (0.24)*** | 0.80 (0.11)*** |  |  | 0.14 (0.16) | 0.09 (0.07) |
| Technical/sales/clerical |  |  | 0.87 (0.21)*** | 0.42 (0.07)*** |  |  | 0.06 (0.09) | 0.02 (0.04) |
| Others |  |  | -0.16 (0.11) | -0.05 (0.06) |  |  | -0.01 (0.15) | 0.08 (0.06) |
| Minorities |  |  | 0.00 (0.09) | -0.05 (0.04) |  |  | 0.04 (0.10) | -0.02 (0.03) |
| Immigrants |  |  | -0.04 (0.10) | -0.06 (0.04) |  |  | 0.11 (0.16) | 0.00 (0.04) |
| Bargaining agreement |  |  | 0.28 (0.17)* | 0.16 (0.05)*** |  |  | 0.06 (0.22) | -0.02 (0.06) |
| International market |  |  | 0.34 (0.08)*** | 0.12 (0.03)*** |  |  |  |  |
| Foreign owned |  |  | 0.54 (0.09)*** | 0.22 (0.04)*** |  |  |  |  |
| Labour tertiary manufacturing |  |  | -0.39 (0.07)*** | -0.32 (0.04)*** |  |  |  |  |
| Primary product manufacturing |  |  | -0.30 (0.07)*** | -0.17 (0.04)*** |  |  |  |  |
| Secondary product manufacturing |  |  | -0.19 (0.07)*** | -0.12 (0.04)*** |  |  |  |  |
| Capital tertiary manufacturing |  |  | -0.22 (0.09)** | -0.15 (0.04)*** |  |  |  |  |
| Construction |  |  | -0.35 (0.12)*** | -0.18 (0.05)*** |  |  |  |  |
| Transportation |  |  | -0.05 (0.07) | -0.14 (0.04)*** |  |  |  |  |
| Communication |  |  | -0.42 (0.07)*** | -0.24 (0.04)*** |  |  |  |  |
| Retail trade |  |  | -0.68 (0.08)*** | -0.79 (0.05)*** |  |  |  |  |
| Finance |  |  | 0.05 (0.09) | -0.12 (0.05)*** |  |  |  |  |
| Real estate |  |  | -0.40 (0.11)*** | -0.45 (0.05)*** |  |  |  |  |
| Business services |  |  | -0.33 (0.09)*** | -0.32 (0.05)*** |  |  |  |  |
| Education |  |  | -0.31 (0.11)*** | -0.46 (0.06)*** |  |  |  |  |
| Information |  |  | -0.57 (0.11)*** | -0.31 (0.05)*** |  |  |  |  |
| Atlantic |  |  | -0.14 (0.10) | -0.15 (0.04)*** |  |  |  |  |
| Quebec |  |  | -0.13 (0.06)** | -0.19 (0.03)*** |  |  |  |  |
| Alberta |  |  | -0.07 (0.06) | -0.06 (0.03)** |  |  |  |  |
| British Columbia |  |  | -0.12 (0.05)** | -0.03 (0.03) |  |  |  |  |
| Manitoba |  |  | -0.05 (0.09) | -0.17 (0.05)*** |  |  |  |  |
| Saskatchewan |  |  | -0.27 (0.09)*** | -0.22 (0.04)*** |  |  |  |  |
| Year01 | -0.10 (0.06)* | -0.03 (0.03) | -0.13 (0.06)** | -0.07 (0.03)** |  |  |  |  |
| Year03 | -0.19 (0.07)*** | -0.08 (0.04)** | -0.18 (0.07)*** | -0.07 (0.03)** | 0.02 (0.04) | 0.04 (0.02)** | 0.02 (0.04) | 0.04 (0.02)** |
| Year05 | -0.10 (0.06)* | -0.05 (0.03)* | -0.09 (0.06) | -0.04 (0.03) | 0.07 (0.03)** | 0.05 (0.02)*** | 0.08 (0.03)** | 0.05 (0.02)*** |
| LnL*LnL | 0.04 (0.01)*** |  | 0.04 (0.01)*** |  | 0.05 (0.04) |  | 0.05 (0.03) |  |
| LnK*LnK | 0.02 (0.00)*** |  | 0.01 (0.00)*** |  | 0.01 (0.01) |  | 0.01 (0.01) |  |
| LnL*LnK | 0.00 (0.01) |  | 0.00 (0.01) |  | -0.04 (0.02)** |  | -0.04 (0.02)** |  |
| Difference in attendance rate coefficients | 0.19 (0.16) |  | 0.13 (0.15) |  | 0.40 (0.40) |  | 0.42 (0.38) |  |
| Difference in team coefficients | 0.33 (0.19)* |  | 0.18 (0.11) |  | 0.04 (0.08) |  | 0.04 (0.08) |  |

Table C.5. Parameter estimates using complete models

|  | **NLS** | | | | **First differences** | | | |
| --- | --- | --- | --- | --- | --- | --- | --- | --- |
|  | **Production** | **Wage** | **Production** | **Wage** | **Production** | **Wage** | **Production** | **Wage** |
| Constant | 13.13 (0.53)*** | 9.41 (0.07)*** | 13.52 (0.82)*** | 10.46 (0.13)*** |  |  |  |  |
| Log (employment) | 0.75 (0.07)*** | 1.04 (0.01)*** | 0.79 (0.08)*** | 1.08 (0.01)*** | 0.86 (0.28)*** | 0.69 (0.04)*** | 0.89 (0.29)*** | 0.70 (0.04)*** |
| Log (stock) | -0.38 (0.09)*** | 0.05 (0.01)*** | -0.38 (0.12)*** | -0.03 (0.01)*** | -0.24 (0.24) | 0.00 (0.01) | -0.24 (0.24) | -0.01 (0.01) |
| Attendance rate, non-team workers | 0.52 (0.18)*** | 0.39 (0.08)*** | 0.55 (0.18)*** | 0.45 (0.07)*** | 0.31 (0.41) | 0.01 (0.11) | 0.32 (0.39) | 0.01 (0.11) |
| Attendance rate, team workers | 2.67 (1.27)** | 1.81 (0.83)** | 2.19 (1.24)* | 1.41 (0.75)* | 1.84 (1.80) | 0.69 (0.62) | 1.93 (1.77) | 0.69 (0.62) |
| Team | 0.56 (0.14)*** | 0.45 (0.08)*** | 0.23 (0.11)** | 0.10 (0.05)** | 0.07 (0.13) | 0.00 (0.04) | 0.07 (0.13) | 0.00 (0.04) |
| 35 ≤ Age < 55 |  |  | 0.33 (0.11)*** | 0.26 (0.04)*** |  |  | 0.01 (0.06) | 0.05 (0.03)* |
| 55≤ Age |  |  | -0.04 (0.21) | 0.18 (0.06)*** |  |  | -0.03 (0.15) | 0.00 (0.04) |
| Female |  |  | -0.30 (0.07)*** | -0.25 (0.02)*** |  |  | 0.04 (0.11) | -0.02 (0.04) |
| > a bachelor’s degree |  |  | 0.28 (0.15)* | 0.32 (0.08)*** |  |  | -0.16 (0.09)* | 0.05 (0.06) |
| Managers/professionals |  |  | 0.80 (0.23)*** | 0.80 (0.11)*** |  |  | 0.14 (0.16) | 0.10 (0.07) |
| Technical/sales/clerical |  |  | 0.86 (0.20)*** | 0.42 (0.07)*** |  |  | 0.06 (0.09) | 0.02 (0.04) |
| Others |  |  | -0.16 (0.11) | -0.05 (0.06) |  |  | -0.01 (0.15) | 0.08 (0.06) |
| Minorities |  |  | 0.00 (0.09) | -0.05 (0.04) |  |  | 0.04 (0.10) | -0.02 (0.03) |
| Immigrants |  |  | -0.04 (0.09) | -0.06 (0.04) |  |  | 0.11 (0.16) | 0.00 (0.04) |
| Bargaining agreement |  |  | 0.29 (0.17)* | 0.16 (0.05)*** |  |  | 0.06 (0.23) | -0.02 (0.06) |
| International market |  |  | 0.34 (0.08)*** | 0.12 (0.03)*** |  |  |  |  |
| Foreign owned |  |  | 0.54 (0.09)*** | 0.22 (0.04)*** |  |  |  |  |
| Labour tertiary manufacturing |  |  | -0.39 (0.07)*** | -0.32 (0.04)*** |  |  |  |  |
| Primary product manufacturing |  |  | -0.30 (0.07)*** | -0.17 (0.04)*** |  |  |  |  |
| Secondary product manufacturing |  |  | -0.19 (0.07)*** | -0.13 (0.04)*** |  |  |  |  |
| Capital tertiary manufacturing |  |  | -0.22 (0.09)** | -0.15 (0.04)*** |  |  |  |  |
| Construction |  |  | -0.35 (0.12)*** | -0.18 (0.05)*** |  |  |  |  |
| Transportation |  |  | -0.06 (0.07) | -0.14 (0.04)*** |  |  |  |  |
| Communication |  |  | -0.42 (0.07)*** | -0.24 (0.04)*** |  |  |  |  |
| Retail trade |  |  | -0.68 (0.08)*** | -0.80 (0.05)*** |  |  |  |  |
| Finance |  |  | 0.05 (0.09) | -0.12 (0.05)*** |  |  |  |  |
| Real estate |  |  | -0.40 (0.11)*** | -0.45 (0.05)*** |  |  |  |  |
| Business services |  |  | -0.33 (0.09)*** | -0.32 (0.05)*** |  |  |  |  |
| Education |  |  | -0.32 (0.11)*** | -0.46 (0.06)*** |  |  |  |  |
| Information |  |  | -0.57 (0.11)*** | -0.31 (0.05)*** |  |  |  |  |
| Atlantic |  |  | -0.14 (0.10) | -0.15 (0.04)*** |  |  |  |  |
| Quebec |  |  | -0.13 (0.06)** | -0.19 (0.03)*** |  |  |  |  |
| Alberta |  |  | -0.07 (0.06) | -0.06 (0.03)** |  |  |  |  |
| British Columbia |  |  | -0.12 (0.05)** | -0.03 (0.03) |  |  |  |  |
| Manitoba |  |  | -0.05 (0.09) | -0.17 (0.05)*** |  |  |  |  |
| Saskatchewan |  |  | -0.27 (0.09)*** | -0.22 (0.04)*** |  |  |  |  |
| Year01 | -0.10 (0.06)* | -0.03 (0.03) | -0.13 (0.06)** | -0.07 (0.03)** |  |  |  |  |
| Year03 | -0.19 (0.07)*** | -0.08 (0.04)** | -0.18 (0.07)*** | -0.06 (0.03)** | 0.02 (0.04) | 0.04 (0.02)** | 0.02 (0.04) | 0.04 (0.02)** |
| Year05 | -0.10 (0.06)* | -0.05 (0.03)* | -0.08 (0.06) | -0.04 (0.03) | 0.08 (0.03)** | 0.05 (0.02)*** | 0.08 (0.03)** | 0.06 (0.02)*** |
| LnL*LnL | 0.04 (0.01)*** |  | 0.04 (0.01)*** |  | 0.05 (0.04) |  | 0.05 (0.03) |  |
| LnK*LnK | 0.02 (0.00)*** |  | 0.01 (0.00)*** |  | 0.01 (0.01) |  | 0.01 (0.01) |  |
| LnL*LnK | 0.00 (0.01) |  | 0.00 (0.01) |  | -0.04 (0.02)** |  | -0.04 (0.02)** |  |
| Difference in attendance coefficients, non-team workers | 0.14 (0.15) |  | 0.10 (0.16) |  | 0.30 (0.38) |  | 0.31 (0.36) |  |
| Difference in attendance coefficients, team workers | 0.86 (0.80) |  | 0.78 (0.86) |  | 1.15 (1.57) |  | 1.25 (1.55) |  |
| Difference in team coefficients | 0.12 (0.10) |  | 0.13 (0.08) |  | 0.06 (0.12) |  | 0.07 (0.12) |  |

Sub-samples: The impacts of total compensation as the outcome of the wage equation

Table C.6. Comparing Nonlinear Least Squares estimates using payroll or total compensation as the outcome of the wage equation

|  | **Payroll** | | **Total compensation** | |
| --- | --- | --- | --- | --- |
|  | **Production** | **Wage** | **Production** | **Wage** |
| **Small firms^‡^** |  |  |  |  |
| Attendance rate, non-team workers | 0.47 (0.14)*** | 0.44 (0.06)*** | 0.49 (0.14)*** | 0.43 (0.07)*** |
| Attendance rate, team workers | 4.97 (1.87)*** | 2.25 (0.95)** | 5.30 (2.41)** | 2.24 (1.06)** |
| Team | 0.33 (0.18)* | 0.06 (0.06) | 0.39 (0.21)* | 0.09 (0.06) |
| Difference in attendance coefficients, non-team workers | 0.03 (0.12) |  | 0.06 (0.11) |  |
| Difference in attendance coefficients, team workers | 2.72 (1.49)* |  | 3.06 (1.90) |  |
| Difference in team coefficients | 0.27 (0.16)* |  | 0.31 (0.20) |  |
| **Large firms^‡^** |  |  |  |  |
| Attendance rate, non-team workers | 1.32 (0.70)* | 1.08 (0.47)** | 1.33 (0.63)** | 1.15 (0.47)** |
| Attendance rate, team workers | -0.76 (0.73) | -0.33 (0.64) | -0.95 (0.71) | -0.35 (0.63) |
| Team | 0.19 (0.10)* | 0.09 (0.07) | 0.15 (0.08)* | 0.11 (0.07)* |
| Difference in attendance coefficients, non-team workers | 0.24 (0.37) |  | 0.19 (0.38) |  |
| Difference in attendance coefficients, team workers | -0.43 (0.72) |  | -0.60 (0.73) |  |
| Difference in team coefficients | 0.10 (0.07) |  | 0.04 (0.06) |  |
| Odd-year data are used; ^‡^Model adjusted for employment, capital stock, occupation, age, sex, education, race, immigrant, bargaining agreement, international market, foreign owned, region, industry and year;  ^***^p≤0.01; ^**^0.01<p≤0.05; ^*^0.05<p≤0.1 | | | | |

Sub-samples: Applying Levinsohn and Petrin’s approach using intermediate inputs to solve this simultaneity problem

Table C.7. Comparing Nonlinear Least Squares estimates between adding a third-order and a fourth-order polynomial approximation in capital and material inputs

|  | **Adding a third-order polynomial approximation in capital and material inputs** | | **Adding a fourth-order approximation in capital and material inputs** | |
| --- | --- | --- | --- | --- |
|  | **Production** | **Wage** | **Production** | **Wage** |
| **Small firms^‡^** |  |  |  |  |
| Attendance rate, non-team workers | 0.49 (0.14)*** | 0.42 (0.07)*** | 0.49 (0.14)*** | 0.42 (0.07)*** |
| Attendance rate, team workers | 4.74 (2.29)** | 2.04 (1.06)* | 4.65 (2.24)** | 2.04 (1.06)* |
| Team | 0.31 (0.20) | 0.08 (0.06) | 0.29 (0.20) | 0.08 (0.06) |
| Difference in attendance coefficients, non-team workers | 0.07 (0.11) |  | 0.06 (0.11) |  |
| Difference in attendance coefficients, team workers | 2.71 (1.80) |  | 2.61 (1.76) |  |
| Difference in team coefficients | 0.23 (0.19) |  | 0.21 (0.18) |  |
| **Large firms^‡^** |  |  |  |  |
| Attendance rate, non-team workers | 1.32 (0.64)** | 1.12 (0.45)** | 1.33 (0.64)** | 1.12 (0.45)** |
| Attendance rate, team workers | -0.97 (0.71) | -0.44 (0.63) | -1.03 (0.72) | -0.44 (0.63) |
| Team | 0.15 (0.08)* | 0.1 (0.06) | 0.14 (0.08)* | 0.10 (0.06) |
| Difference in attendance coefficients, non-team workers | 0.20 (0.38) |  | 0.21 (0.39) |  |
| Difference in attendance coefficients, team workers | -0.53 (0.76) |  | -0.59 (0.76) |  |
| Difference in team coefficients | 0.04 (0.07) |  | 0.03 (0.07) |  |
| Odd-year data are used; ^‡^Model adjusted for employment, capital stock, occupation, age, sex, education, race, immigrant, bargaining agreement, international market, foreign owned, industry and year;  ^***^p≤0.01; ^**^0.01<p≤0.05; ^*^0.05<p≤0.1 | | | | |

Full sample: The impacts of the equal absence rate assumption

Table C.8. Comparing Nonlinear Least Squares estimates with and without assuming equal absence rates between team workers and non-team workers

|  | **Equal absence rates** | | **Unequal absence rates** | |
| --- | --- | --- | --- | --- |
|  | **Production** | **Wage** | **Production** | **Wage** |
| Baseline controls^†^ |  |  |  |  |
| Attendance rate, non-team workers | 0.37 (0.12)*** | 0.38 (0.07)*** | 0.37 (0.12)*** | 0.38 (0.07)*** |
| Attendance rate, team workers | 2.78 (1.44)* | 1.83 (0.84)** | 3.28 (1.86)* | 1.76 (0.60)*** |
| Team | 0.75 (0.17)*** | 0.45 (0.08)*** | 0.76 (0.17)*** | 0.45 (0.08)*** |
| Difference in attendance coefficients, non-team workers | -0.01 (0.10) |  | -0.01 (0.09) |  |
| Difference in attendance coefficients, team workers | 0.95 (0.95) |  | 1.52 (1.50) |  |
| Difference in team coefficients | 0.30 (0.12)** |  | 0.30 (0.13)** |  |
| All controls^‡^ |  |  |  |  |
| Attendance rate, non-team workers | 0.43 (0.13)*** | 0.45 (0.07)*** | 0.43 (0.12)*** | 0.45 (0.06)*** |
| Attendance rate, team workers | 2.38 (1.40)* | 1.43 (0.75)* | 3.35 (1.82)* | 1.49 (0.58)** |
| Team | 0.32 (0.12)** | 0.10 (0.05)** | 0.34 (0.12)*** | 0.10 (0.05)** |
| Difference in attendance coefficients, non-team workers | -0.02 (0.10) |  | -0.02 (0.10) |  |
| Difference in attendance coefficients, team workers | 0.95 (1.00) |  | 1.87 (1.51) |  |
| Difference in team coefficients | 0.21 (0.10)** |  | 0.23 (0.10)** |  |
| Odd-year data are used; ^†^Model adjusted for employment, capital stock, and years; ^‡^Model adjusted for employment, capital stock, occupation, age, sex, education, race, immigrant, bargaining agreement, international market, foreign owned, region, industry and year;  ^***^p≤0.01; ^**^0.01<p≤0.05; ^*^0.05<p≤0.1 | | | | |

Table C.9. Comparing first differences estimates with and without assuming equal absence rates between team workers and non-team workers

|  | **Equal absence rates** | | **Unequal absence rates** | |
| --- | --- | --- | --- | --- |
|  | **Production** | **Wage** | **Production** | **Wage** |
| Baseline controls^†^ |  |  |  |  |
| Attendance rate, non-team workers | 0.27 (0.39) | 0.01 (0.11) | 0.20 (0.36) | 0.00 (0.10) |
| Attendance rate, team workers | 2.72 (2.08) | 0.71 (0.62) | 2.14 (1.82) | 0.45 (0.57) |
| Team | 0.13 (0.17) | 0.00 (0.04) | 0.13 (0.17) | 0.00 (0.04) |
| Difference in attendance coefficients, non-team workers | 0.27 (0.35) |  | 0.20 (0.33) |  |
| Difference in attendance coefficients, team workers | 2.02 (1.85) |  | 1.69 (1.57) |  |
| Difference in team coefficients | 0.13 (0.16) |  | 0.13 (0.16) |  |
| All controls^‡^ |  |  |  |  |
| Attendance rate, non-team workers | 0.29 (0.37) | 0.01 (0.11) | 0.21 (0.34) | 0.00 (0.10) |
| Attendance rate, team workers | 2.73 (1.92) | 0.71 (0.64) | 2.17 (1.84) | 0.45 (0.60) |
| Team | 0.14 (0.18) | 0.00 (0.04) | 0.13 (0.18) | 0.00 (0.04) |
| Difference in attendance coefficients, non-team workers | 0.28 (0.33) |  | 0.21 (0.31) |  |
| Difference in attendance coefficients, team workers | 2.02 (1.70) |  | 1.72 (1.58) |  |
| Difference in team coefficients | 0.14 (0.17) |  | 0.14 (0.16) |  |
| Odd-year data are used; ^†^Model adjusted for employment, capital stock, and years; ^‡^Model adjusted for employment, capital stock, occupation, age, sex, education, race, immigrant, bargaining agreement, and year; Standard error in the bracket;  ^***^p≤0.01; ^**^0.01<p≤0.05; ^*^0.05<p≤0.1 | | | | |

Sub-samples: The impacts of the equal absence rate assumption

Table C.10. Comparing Nonlinear Least Squares estimates with and without assuming equal absence rates between team workers and non-team workers

|  | **Equal absence rates** | | **Unequal absence rates** | |
| --- | --- | --- | --- | --- |
|  | **Production** | **Wage** | **Production** | **Wage** |
| **Small firms^‡^** |  |  |  |  |
| Attendance rate, non-team workers | 0.47 (0.14)*** | 0.44 (0.06)*** | 0.47 (0.14)*** | 0.44 (0.06)*** |
| Attendance rate, team workers | 4.97 (1.87)*** | 2.25 (0.95)** | 5.91 (1.63)*** | 1.96 (1.03)* |
| Team | 0.33 (0.18)* | 0.06 (0.06) | 0.36 (0.18)* | 0.06 (0.05) |
| Difference in attendance coefficients, non-team workers | 0.03 (0.12) |  | 0.03 (0.12) |  |
| Difference in attendance coefficients, team workers | 2.72 (1.49)* |  | 3.96 (1.34)*** |  |
| Difference in team coefficients | 0.27 (0.16)* |  | 0.30 (0.16)* |  |
| **Large firms^‡^** |  |  |  |  |
| Attendance rate, non-team workers | 1.32 (0.70)* | 1.08 (0.47)** | 1.13 (0.66)* | 0.85 (0.44)* |
| Attendance rate, team workers | -0.76 (0.73) | -0.33 (0.64) | 0.00 (0.08) | 0.54 (0.49) |
| Team | 0.19 (0.10)* | 0.09 (0.07) | 0.22 (0.09)** | 0.12 (0.07)* |
| Difference in attendance coefficients, non-team workers | 0.24 (0.37) |  | 0.28 (0.34) |  |
| Difference in attendance coefficients, team workers | -0.43 (0.72) |  | -0.54 (0.52) |  |
| Difference in team coefficients | 0.10 (0.07) |  | 0.10 (0.07) |  |
| Odd-year data are used; ^‡^Model adjusted for employment, capital stock, occupation, age, sex, education, race, immigrant, bargaining agreement, international market, foreign owned, region, industry and year;  ^***^p≤0.01; ^**^0.01<p≤0.05; ^*^0.05<p≤0.1 | | | | |

Table C.11. Comparing first differences estimates with and without assuming equal absence rates between team workers and non-team workers

|  | **Equal absence rates** | | **Unequal absence rates** | |
| --- | --- | --- | --- | --- |
|  | **Production** | **Wage** | **Production** | **Wage** |
| **Small firms^‡^** |  |  |  |  |
| Attendance rate, non-team workers | 0.49 (0.47) | 0.02 (0.12) | 0.20 (0.43) | 0.00 (0.11) |
| Attendance rate, team workers | 4.36 (2.23)* | 1.25 (1.07) | 3.44 (2.07)* | 0.78 (0.85) |
| Team | 0.07 (0.28) | 0.08 (0.07) | 0.09 (0.28) | 0.07 (0.07) |
| Difference in attendance coefficients, non-team workers | 0.47 (0.43) |  | 0.20 (0.41) |  |
| Difference in attendance coefficients, team workers | 3.11 (2.04) |  | 2.66 (1.92) |  |
| Difference in team coefficients | -0.01 (0.25) |  | 0.02 (0.25) |  |
| **Large firms^‡^** |  |  |  |  |
| Attendance rate, non-team workers | 0.19 (0.36) | 0.16 (0.18) | 0.40 (0.31) | 0.09 (0.16) |
| Attendance rate, team workers | -0.73 (1.40) | 0.02 (0.33) | -0.10 (0.07) | 0.39 (0.26) |
| Team | 0.05 (0.11) | -0.06 (0.05) | 0.06 (0.10) | -0.05 (0.05) |
| Difference in attendance coefficients, non-team workers | 0.03 (0.32) |  | 0.31 (0.26) |  |
| Difference in attendance coefficients, team workers | -0.74 (1.38) |  | -0.48 (0.30) |  |
| Difference in team coefficients | 0.11 (0.12) |  | 0.11 (0.11) |  |
| Odd-year data are used; ^‡^Model adjusted for employment, capital stock, occupation, age, sex, education, race, immigrant, bargaining agreement, and year; Standard error in the bracket;  ^***^p≤0.01; ^**^0.01<p≤0.05; ^*^0.05<p≤0.1 | | | | |

Sub-samples: Different estimation methods using complete models

Table C.12. Comparing Nonlinear Least Squares estimates, first difference estimates using odd-year data, and first difference estimates between odd-even years

|  | **Nonlinear least squares** | | **First differences** | | **First differences (odd-even years)** | |
| --- | --- | --- | --- | --- | --- | --- |
|  | **Production** | **Wage** | **Production** | **Wage** | **Production** | **Wage** |
| **Small firms^‡^** |  |  |  |  |  |  |
| Attendance rate, non-team workers | 0.47 (0.14)*** | 0.44 (0.06)*** | 0.49 (0.47) | 0.02 (0.12) | -0.31 (0.38) | -0.11 (0.11) |
| Attendance rate, team workers | 4.97 (1.87)*** | 2.25 (0.95)** | 4.36 (2.23)* | 1.25 (1.07) | 1.44 (3.06) | 1.04 (0.48)* |
| Team | 0.33 (0.18)* | 0.06 (0.06) | 0.07 (0.28) | 0.08 (0.07) | 0.11 (0.27) | 0.03 (0.05) |
| Difference in attendance coefficients, non-team workers | 0.03 (0.12) |  | 0.47 (0.43) |  | -0.20 (0.34) |  |
| Difference in attendance coefficients, team workers | 2.72 (1.49)* |  | 3.11 (2.04) |  | 0.40 (3.1) |  |
| Difference in team coefficients | 0.27 (0.16)* |  | -0.01 (0.25) |  | 0.08 (0.28) |  |
| **Large firms^‡^** |  |  |  |  |  |  |
| Attendance rate, non-team workers | 1.32 (0.70)* | 1.08 (0.47)** | 0.19 (0.36) | 0.16 (0.18) | 0.26 (0.21) | 0.18 (0.11) |
| Attendance rate, team workers | -0.76 (0.73) | -0.33 (0.64) | -0.73 (1.40) | 0.02 (0.33) | 0.65 (0.71) | 0.71 (0.19)* |
| Team | 0.19 (0.10)* | 0.09 (0.07) | 0.05 (0.11) | -0.06 (0.05) | 0.05 (0.09) | 0.02 (0.02) |
| Difference in attendance coefficients, non-team workers | 0.24 (0.37) |  | 0.03 (0.32) |  | 0.08 (0.19) |  |
| Difference in attendance coefficients, team workers | -0.43 (0.72) |  | -0.74 (1.38) |  | -0.05 (0.61) |  |
| Difference in team coefficients | 0.10 (0.07) |  | 0.11 (0.12) |  | 0.03 (0.08) |  |
| ^‡^Nonlinear Least Squares estimates adjusted for employment, capital stock, occupation, age, sex, education, race, immigrant, bargaining agreement, international market, foreign owned, region, industry and year; First differences estimates using odd years only adjusted for employment, capital stock, occupation, age, sex, education, race, immigrant, bargaining agreement, and year; First differences (odd-even years) estimates using common workplaces and employees between 1999 to 2000, 2001 to 2002, and 2003 to 2004 adjusted for employment, capital stock, age, occupation, bargaining agreement, and year; Standard error in the bracket; ^***^p≤0.01; ^**^0.01<p≤0.05; ^*^0.05<p≤0.1 | | | | | | |

References

1. Hellerstein, J.K., Neumark, Troske, K.R.: Wages, Productivity, and Worker Characteristics: Evidence from Plant‐Level Production Functions and Wage Equations. J. Labor Econ. 17, 409–446 (1999).

2. Hellerstein, J.K., Neumark, D.: Sex, Wages, and Productivity: An Empirical Analysis of Israeli Firm-Level Data. Int. Econ. Rev. 40, 95–123 (1999).

3. Zhang, W., Sun, H., Woodcock, S., Anis, A.: Illness related wage and productivity losses: Valuing “presenteeism.” Soc. Sci. Med. 1982. 147, 62–71 (2015).

4. van Ours, J.C., Stoeldraijer, L.: Age, wage and productivity. IZA (2010).

5. Aubert, P., Crépon, B.: La productivité des salariés âgés: une tentative d’estimation. Économie Stat. 368, 95–119 (2003).

6. Crépon, B., Deniau, N., Pérez-Duarte, S.: Wages, Productivity and Worker Characteristics : A French Perspective. Centre de Recherche en Economie et Statistique (2003).

7. Levinsohn, J., Petrin, A.: Estimating Production Functions Using Inputs to Control for Unobservables. Rev. Econ. Stud. 70, 317–341 (2003).

8. Petrin, A., Poi, B.P., Levinsohn, J.: Production Function Estimation in STATA using Inputs to Control for Unobservables. Stata J. 4, 113–123 (2004).
